# Supplementary material for: Generation of amine dehydrogenases with increased catalytic performance and substrate scope from ε-deaminating L-Lysine dehydrogenase
Source: Nat Commun. 2019 Aug 16;10:3717. doi: 10.1038/s41467-019-11509-x (PMC6697735; doi:10.1038/s41467-019-11509-x)
Supplement: Supplementary file 1 — Supplementary Information [file 41467_2019_11509_MOESM1_ESM.pdf]

Supplementary Information for:

# Generation of Amine Dehydrogenases with Increased Catalytic Performance and Substrate Scope from ε-deaminating *L*-Lysine Dehydrogenase

Vasilis Tseliou[1], Tanja Knaus[1,*], Marcelo F. Masman[1], Maria L. Corrado[1], Francesco G. Mutti[1,*]

*Van't Hoff Institute for Molecular Sciences, University of Amsterdam, Science Park 904, 1098 XH Amsterdam, The Netherlands.*

* Corresponding authors: t.knaus@uva.nl, f.mutti@uva.nl

## Table of Contents

**Supplementary Figures** 2

**Supplementary Tables** 13

**Supplementary Methods** 27

**Supplementary Notes** 45

**Supplementary References** 46

## Supplementary Figures

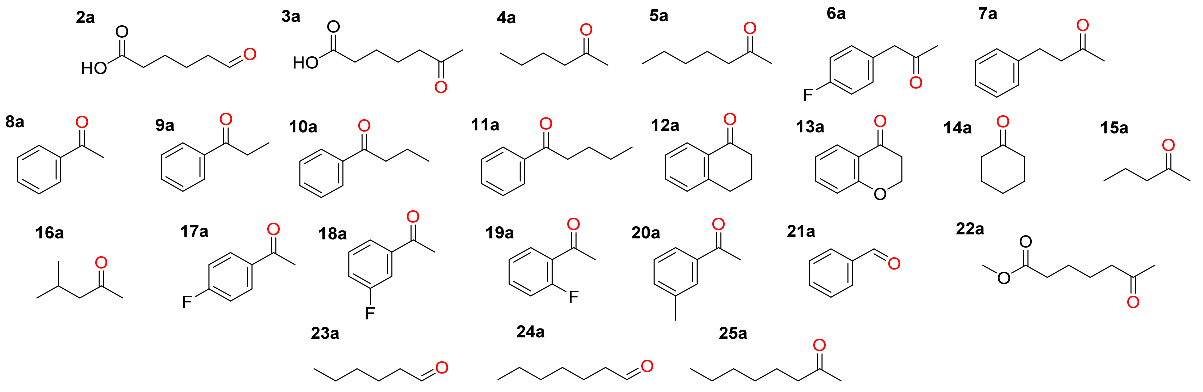


**Supplementary Figure 1**. **List of prochiral ketones and aldehydes tested in this study**. Compounds that have been used as substrates in the biocatalytic reactions performed by the engineered amine dehydrogenases.

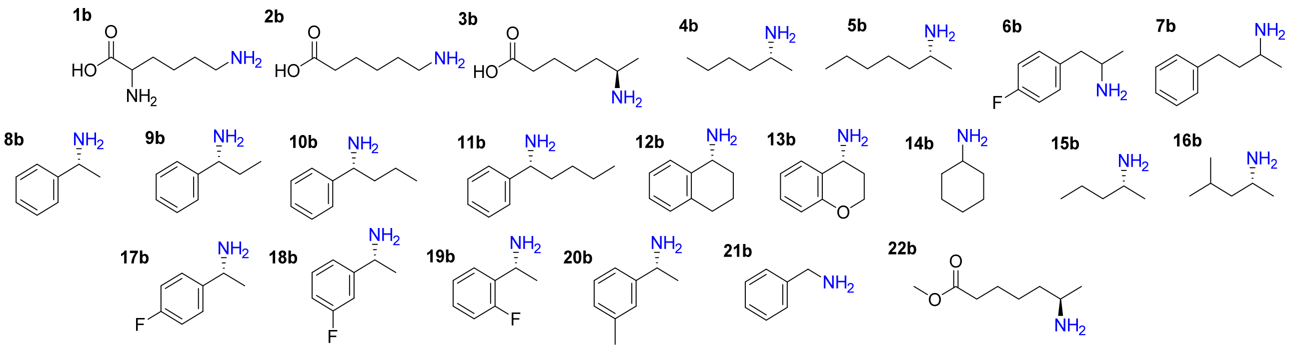


**Supplementary Figure 2**. **List of products obtained in this study**. Compounds that have been identified as products in the biocatalytic reactions performed by the engineered amine dehydrogenases.

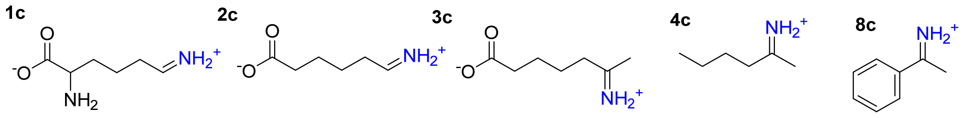


**Supplementary Figure 3**. **List of iminium intimidates used in this study.** Compound listed above mainly used for computational studies.

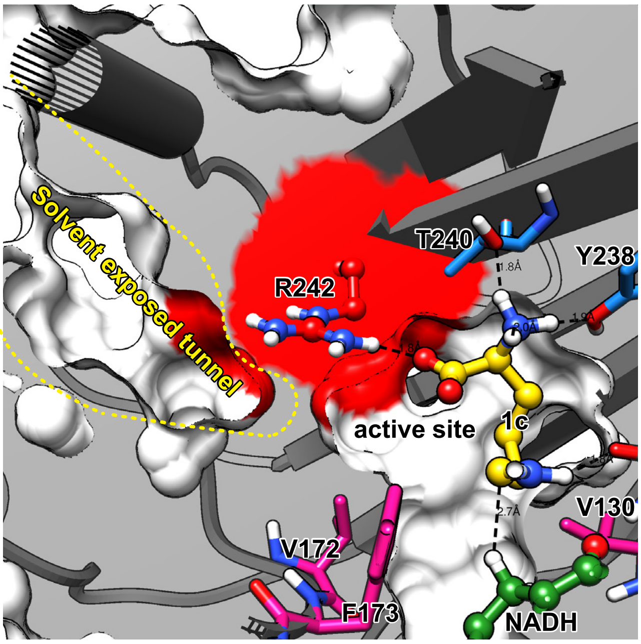


**Supplementary Figure 4. Residue arginine 242 is the gate keeper of the active site**. A view into the active site of the single mutant F173A shows that the residue R242 could act as a gate for the accessibility of the substrate. In this figure R242 is depicted in red with a red-coloured surface (radius of 3 Å) around this residue. The natural substrate **1c** and the coenzyme (NADH) are also shown.

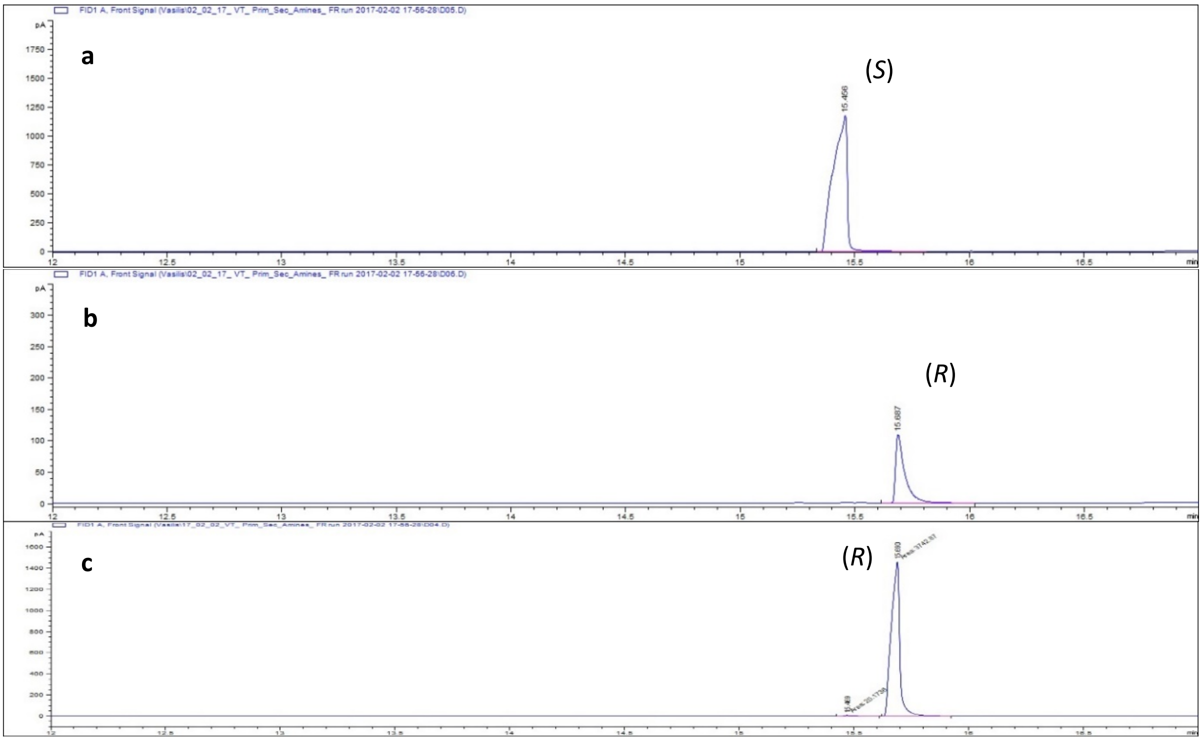


**Supplementary Figure 5. GC-FID chromatograms for the determination of the absolute configuration of 4b.** (a) reference *S*-configured amine. (b) reference *R*-configured amine. (c) Biocatalytic reaction catalysed by LE-AmDH-v1. The areas obtained from the reaction with LE-AmDH-v1 were 3743 for the *R*- and 20 for the *S*-configured amines.

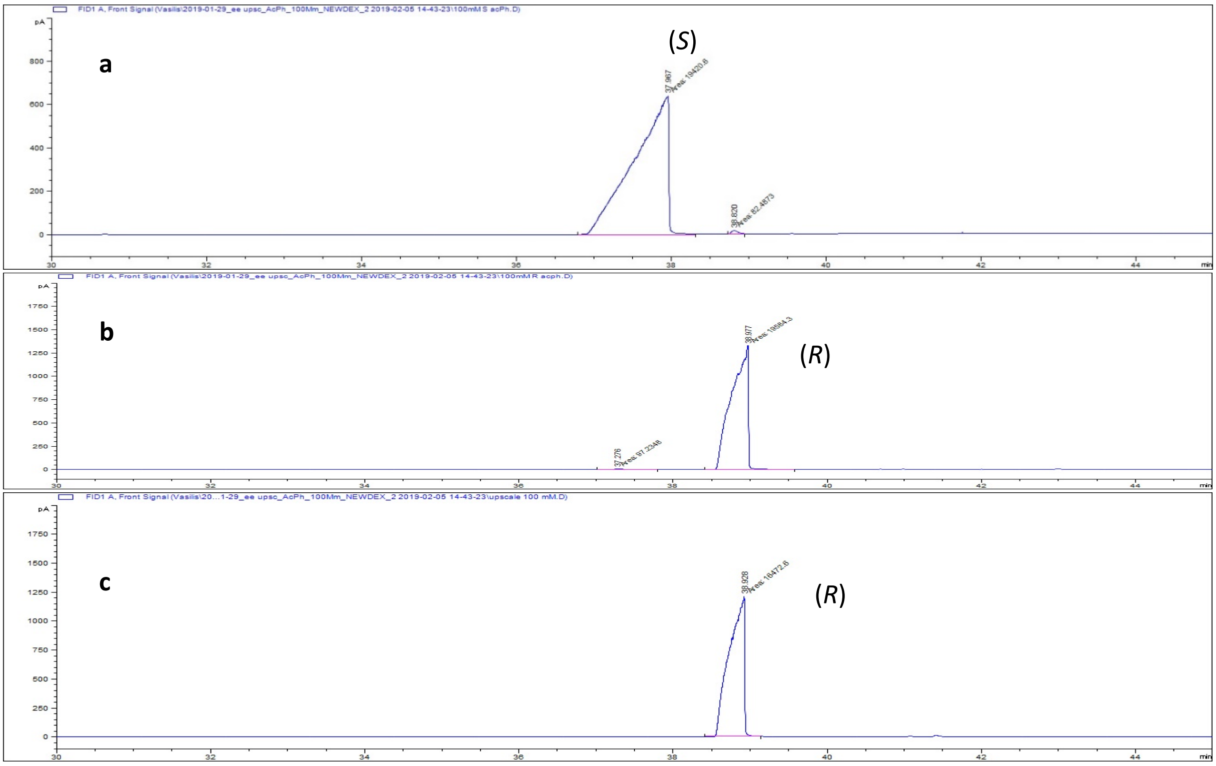


**Supplementary Figure 6**. **GC-FID chromatograms for the determination of the absolute configuration of 8b.** (a) reference S- configured amine. (b) reference *R*-configured amine. (c) Biocatalytic reaction catalysed by LE-AmDH-v1. The areas obtained from the reaction with LE-AmDH-v1 were 16473 for the *R*- and 0 for the *S*-configured amines.

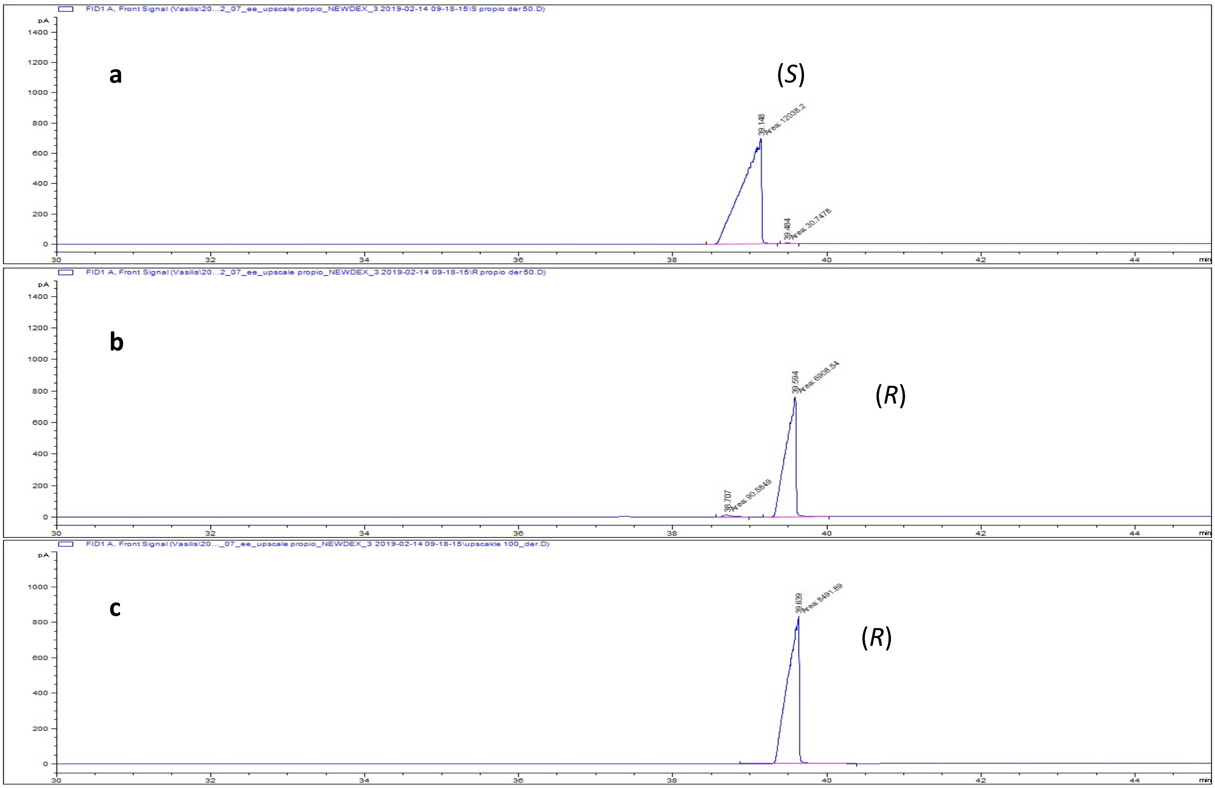


**Supplementary Figure 7**. **GC-FID chromatograms for the determination of the absolute configuration of 9b.** (a) reference *S*-configured amine. (b) reference *R*-configured amine. (c) Biocatalytic reaction catalysed by LE-AmDH-v1. The areas obtained from the reaction with LE-AmDH-v1 were 8492 for the *R*- and 0 for the *S*-configured amines.

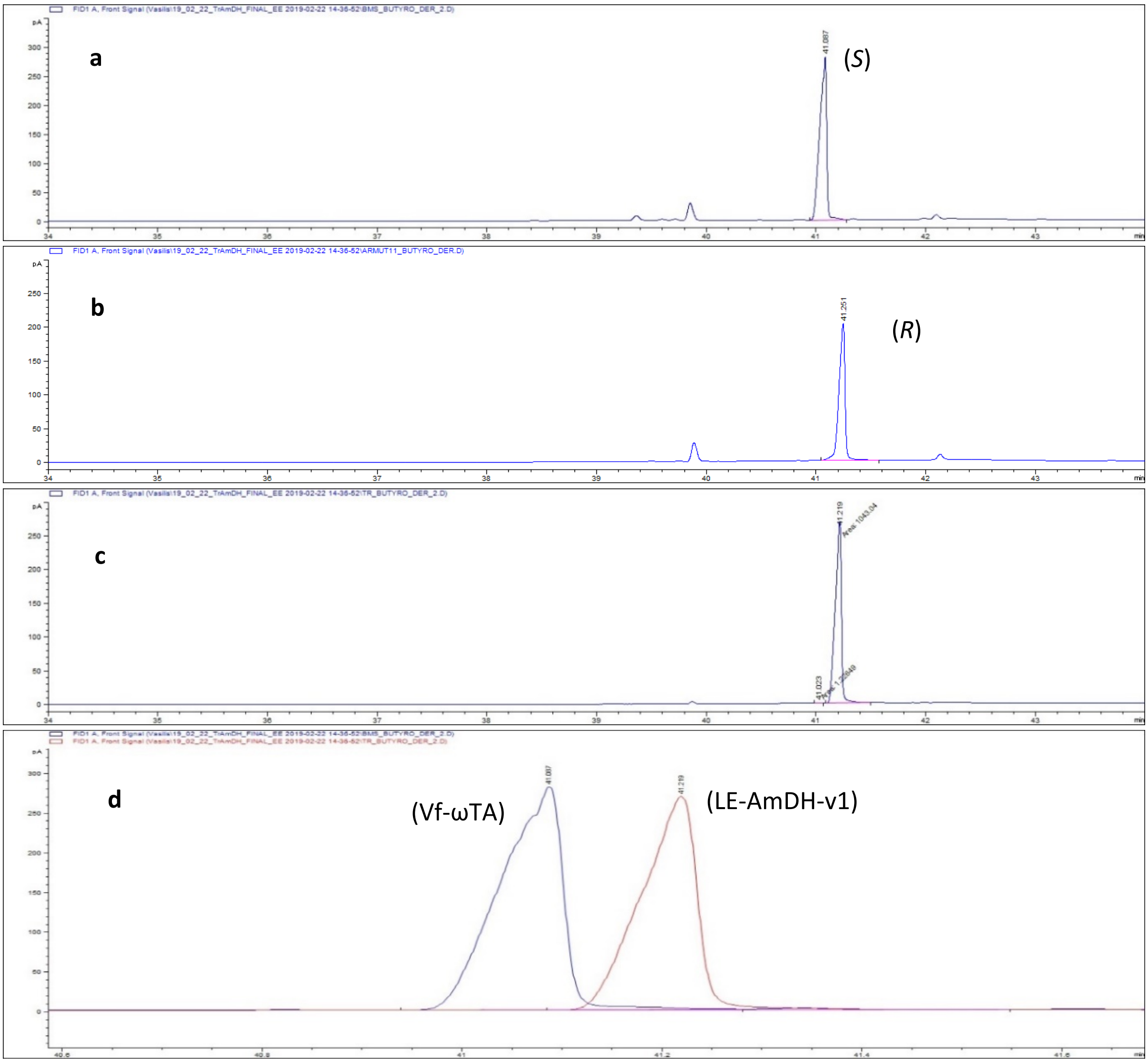


**Supplementary Figure 8**. **GC-FID chromatograms for the determination of the absolute configuration of 10b.** (a) reference *S*-configured amine synthetized by the *S*-selective Vf-ωTA. (b) reference *R*-configured amine synthetized by the *R*-selective AsRmut11-ωTA. (c) Biocatalytic reaction catalysed by LE-AmDH-v1. The areas obtained from the reaction with LE-AmDH-v1 were 1045 for the *R*- and 1.2 for the *S*-configured amines. (d) Overlay of the chromatograms obtained by Vf- ωTA and LE-AmDH-v1.

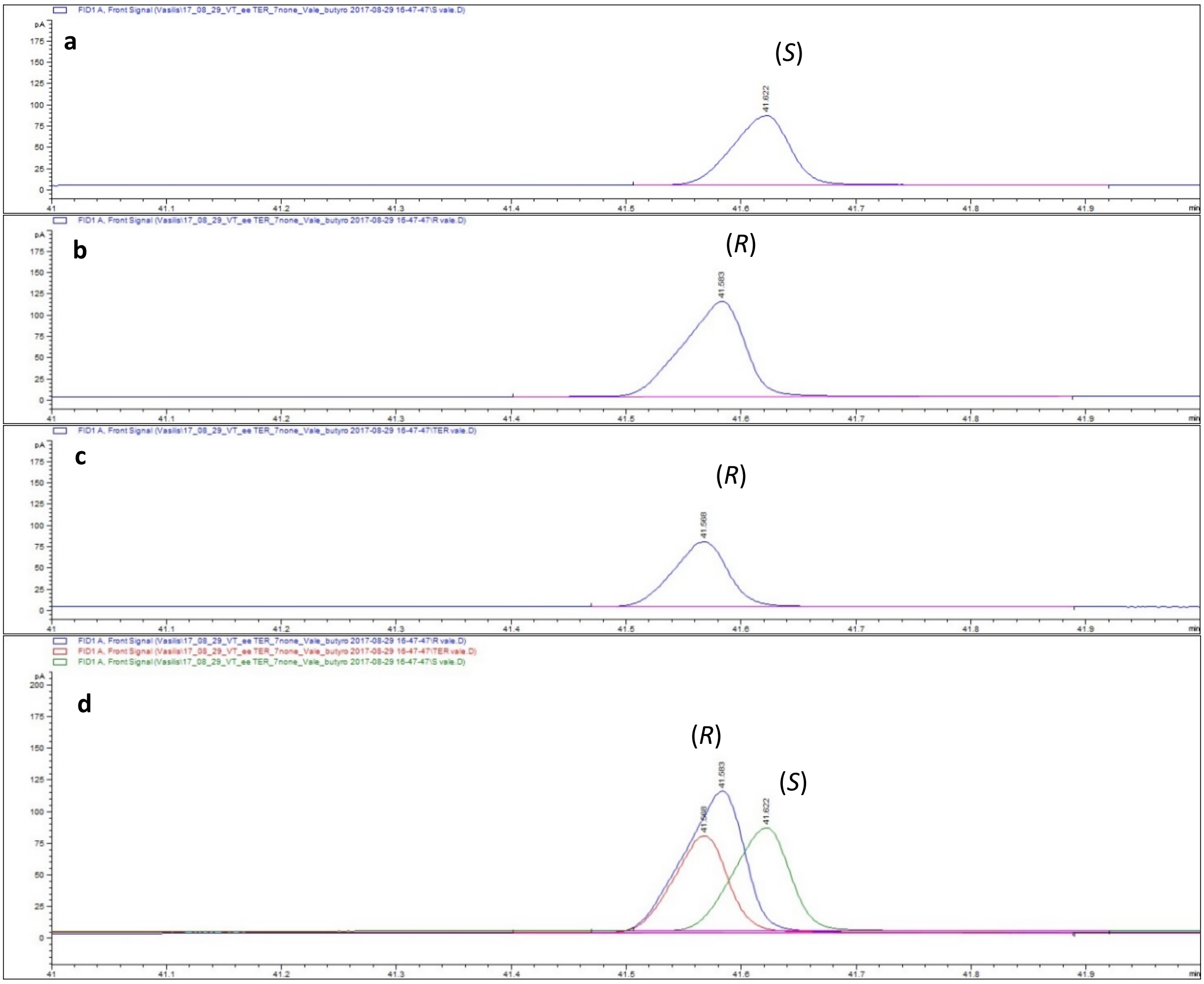


**Supplementary Figure 9**. **GC-FID chromatograms for the determination of the absolute configuration of 11b.** (a) reference *S*-configured amine synthetized by the *S* selective Vf-ωTA. (b) reference *R*-configured amine synthetized by the *R* selective AsRmut11-ωTA. (c) Biocatalytic reaction catalysed by LE-AmDH-v1. The areas obtained from the reaction with LE-AmDH-v1 were 1045 for the *R*- and 1.2 for the *S*-configured amines. (d) Overlay of the chromatographs obtained by Vf-ωTA (green), AsRmut11-ωTA (blue) and LE-AmDH-v1 (red).

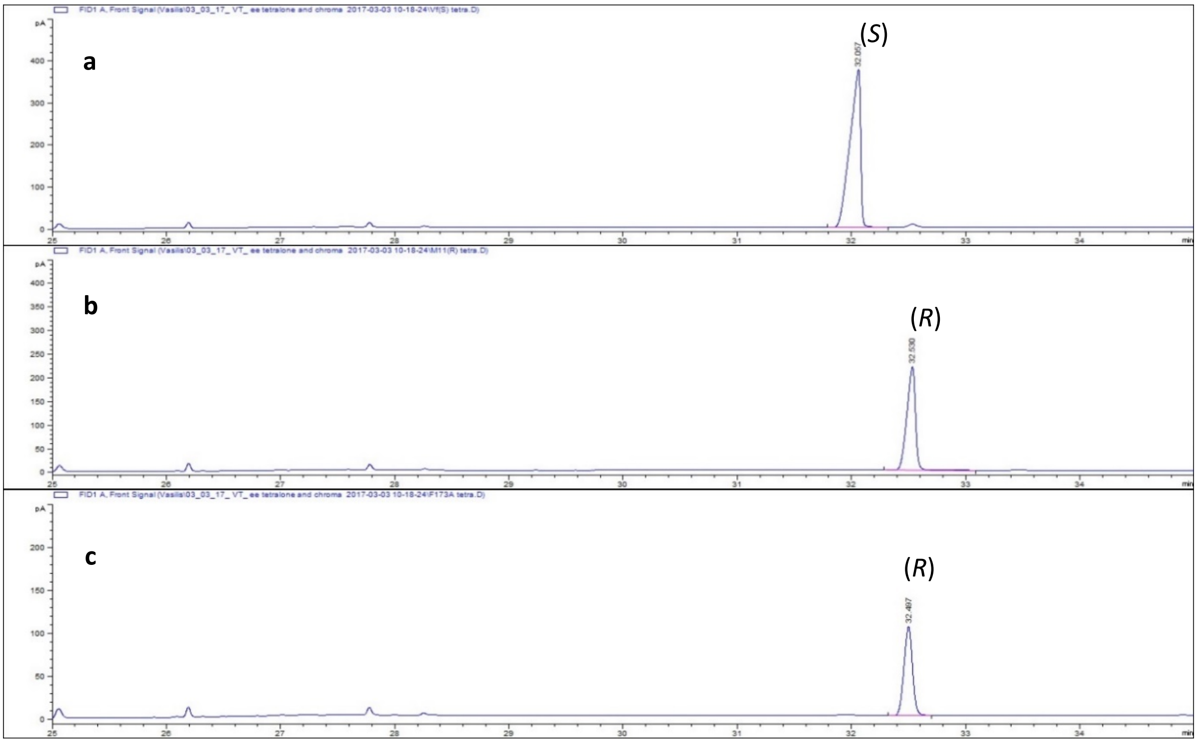


**Supplementary Figure 10**. **GC-FID chromatograms for the determination of the absolute configuration of 12b.** (a) reference *S*-configured amine synthetized by the *S*-selective Vf-ωTA. (b) reference *R*-configured amine synthetized by the *R*-selective AsRmut11-ωTA. (c) Biocatalytic reaction catalysed by LE-AmDH-v1. The areas obtained from the reaction with LE-AmDH-v1 were 531 for the *R*- and 0 for the *S*-configured amines.

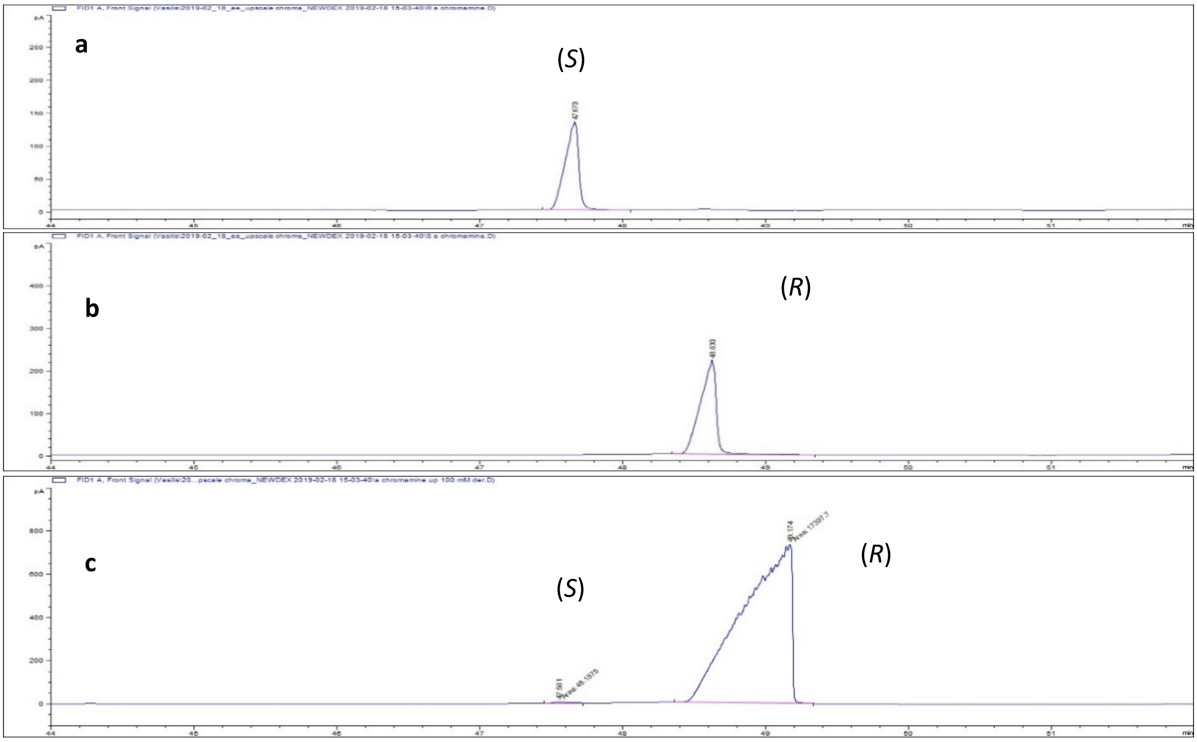


**Supplementary Figure 11**. **GC-FID chromatograms for the determination of the absolute configuration of 13b.** (a) reference *S*-configured amine synthetized by the *S*-selective Vf-ωTA. (b) reference *R*-configured amine synthetized by the *R*-selective AsRmut11-ωTA. (c) Biocatalytic reaction catalysed by LE-AmDH-v1. The areas obtained from the reaction with LE-AmDH-v1 were 17397 for the *R*- and 48 for the *S*- configured amines.

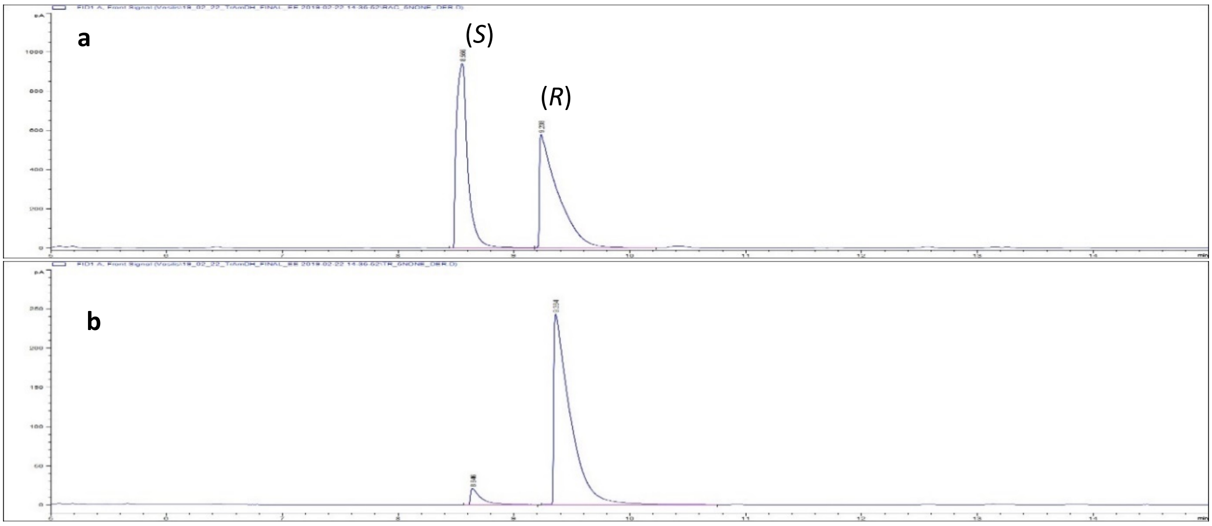


**Supplementary Figure 12. GC-FID chromatograms for the determination of the absolute configuration of 15b.** (a) reference *rac*-amine. (b) Biocatalytic reaction catalysed by LE-AmDH-v1. The areas obtained from the reaction with LE-AmDH-v1 were 2389 for the *R*- and 134 for the *S*-configured amines.

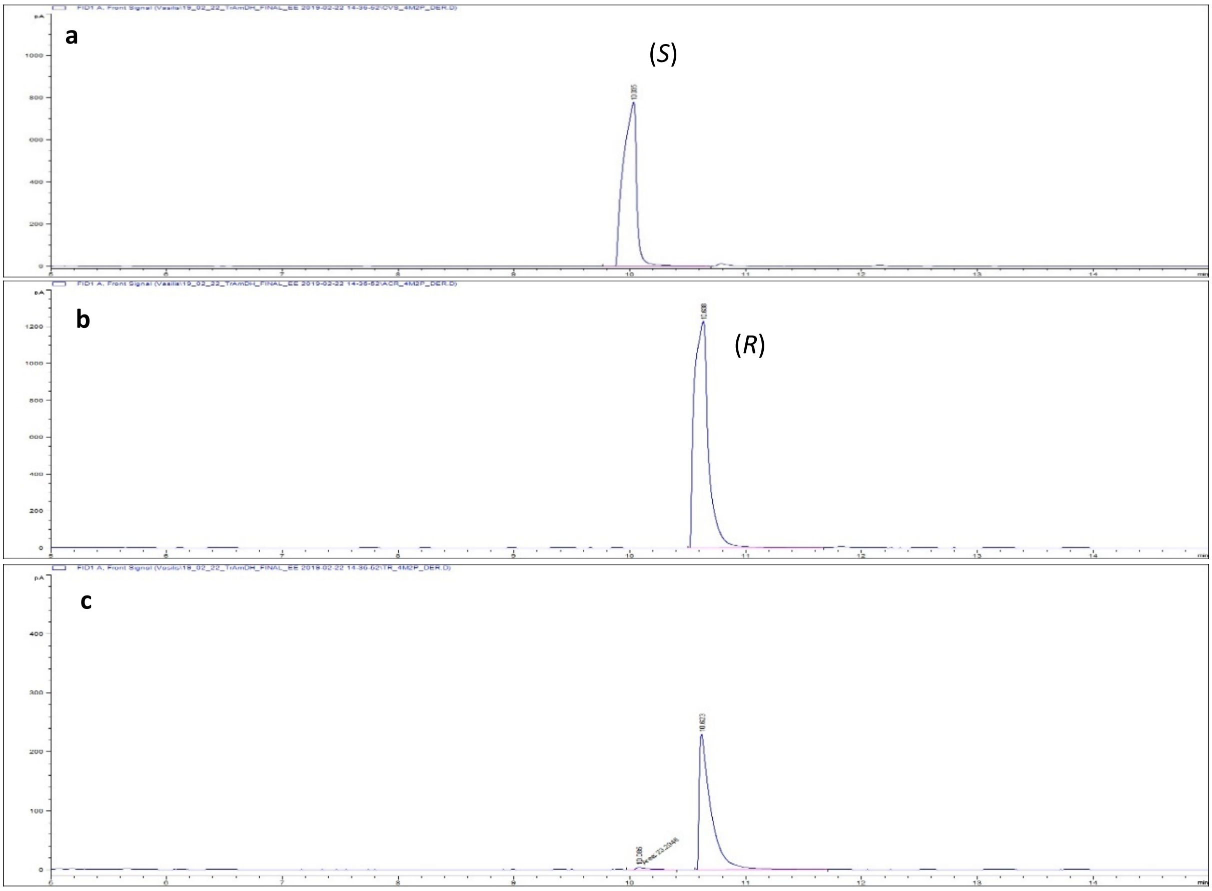


**Supplementary Figure 13**. **GC-FID chromatograms for the determination of the absolute configuration of 16b.** (a) reference *S*-configured amine synthetized by the *S*-selective Cv-ωTA. (b) reference *R*-configured amine synthetized by the *R*-selective Hn-ωTA. (c) Biocatalytic reaction catalysed by LE-AmDH-v1. The areas obtained from the reaction with LE-AmDH-v1 were 1611 for the *R*- and 23 for the *S*-configured amines.

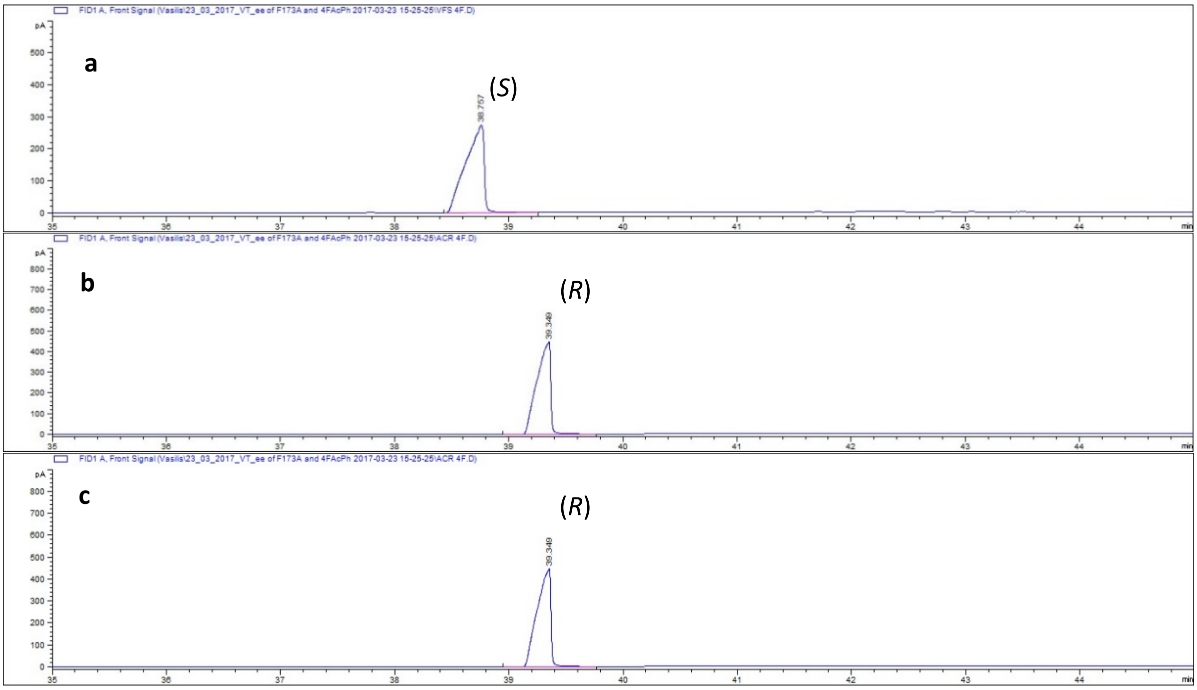


**Supplementary Figure 14**. **GC-FID chromatograms for the determination of the absolute configuration of 17b.** (a) reference *S*-configured amine synthetized by the *S*-selective Cv-ωTA. (b) reference *R*-configured amine synthetized by the *R*-selective As-ωTA. (c) Biocatalytic reaction catalysed by LE-AmDH-v1. The areas obtained from the reaction with LE-AmDH-v1 were 2394 for the *R*- and 0 for the *S*-configured amines.

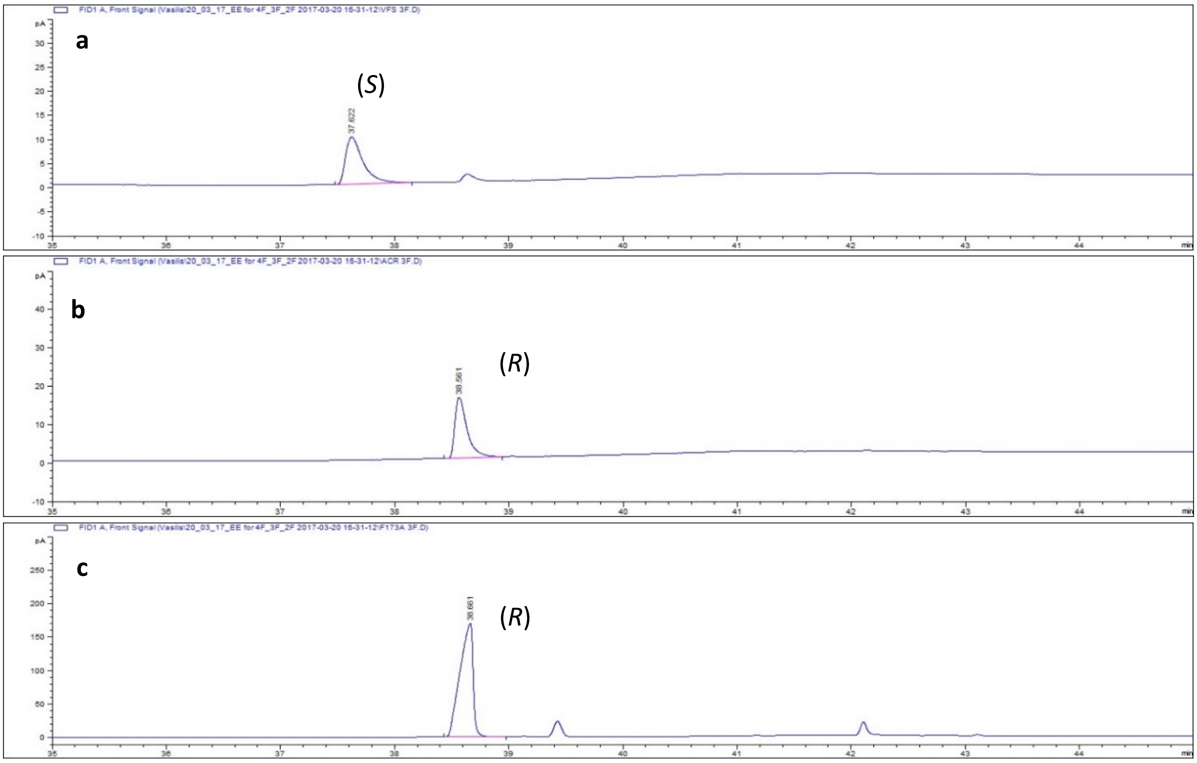


**Supplementary Figure 15**. **GC-FID chromatograms for the determination of the absolute configuration of 18b.** (a) reference *S*-configured amine synthetized by the *S*-selective Vf-ωTA. (b) reference *R*-configured amine synthetized by the *R*-selective As-ωTA. (c) Biocatalytic reactions catalysed by LE-AmDH-v1. The areas obtained from the reaction with LE-AmDH-v1 were 1349 for the *R*- and 0 for the *S*-configured amines.

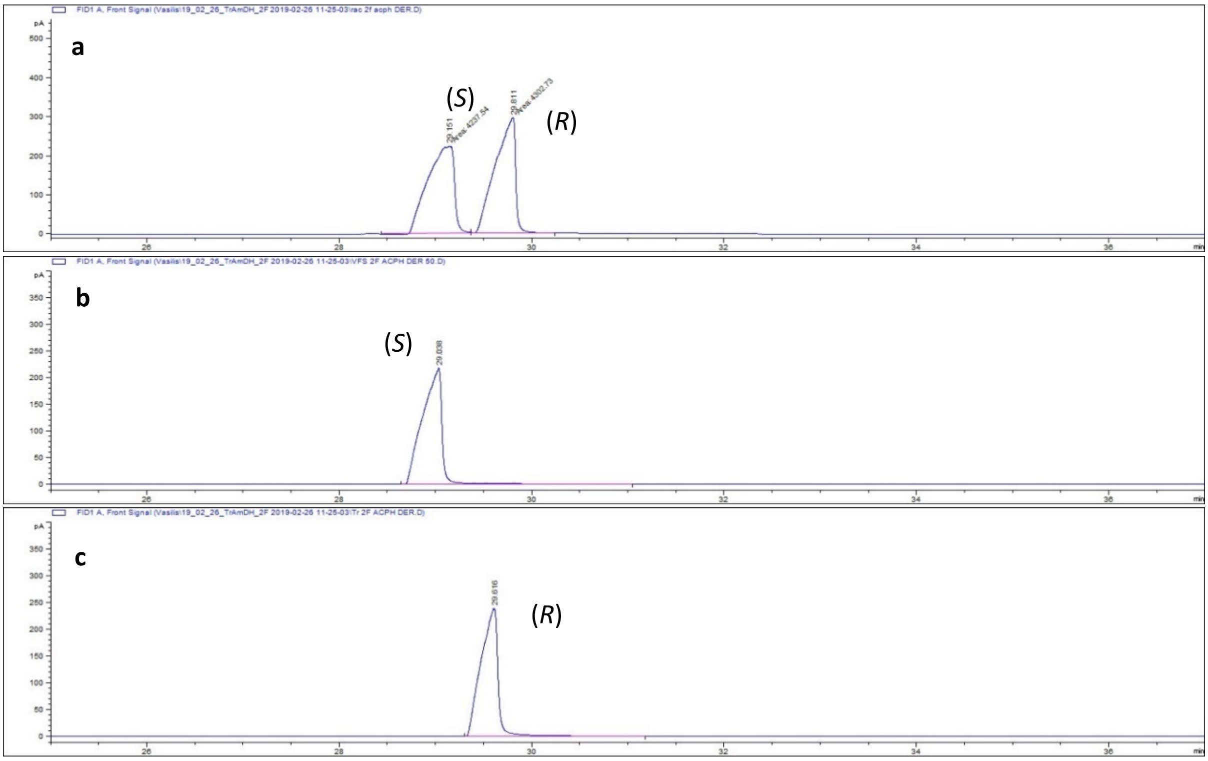


**Supplementary Figure 16**. **GC-FID chromatograms for the determination of the absolute configuration of 19b.** (a) reference *rac*-amine. (b) reference *S*-configured amine synthetized by the *R*-selective Vf-ωTA. (c) Biocatalytic reaction catalysed by LE-AmDH-v1. The areas obtained from the reaction with LE-AmDH-v1 were 2867 for the *R*- and 0 for the *S*-configured amines.

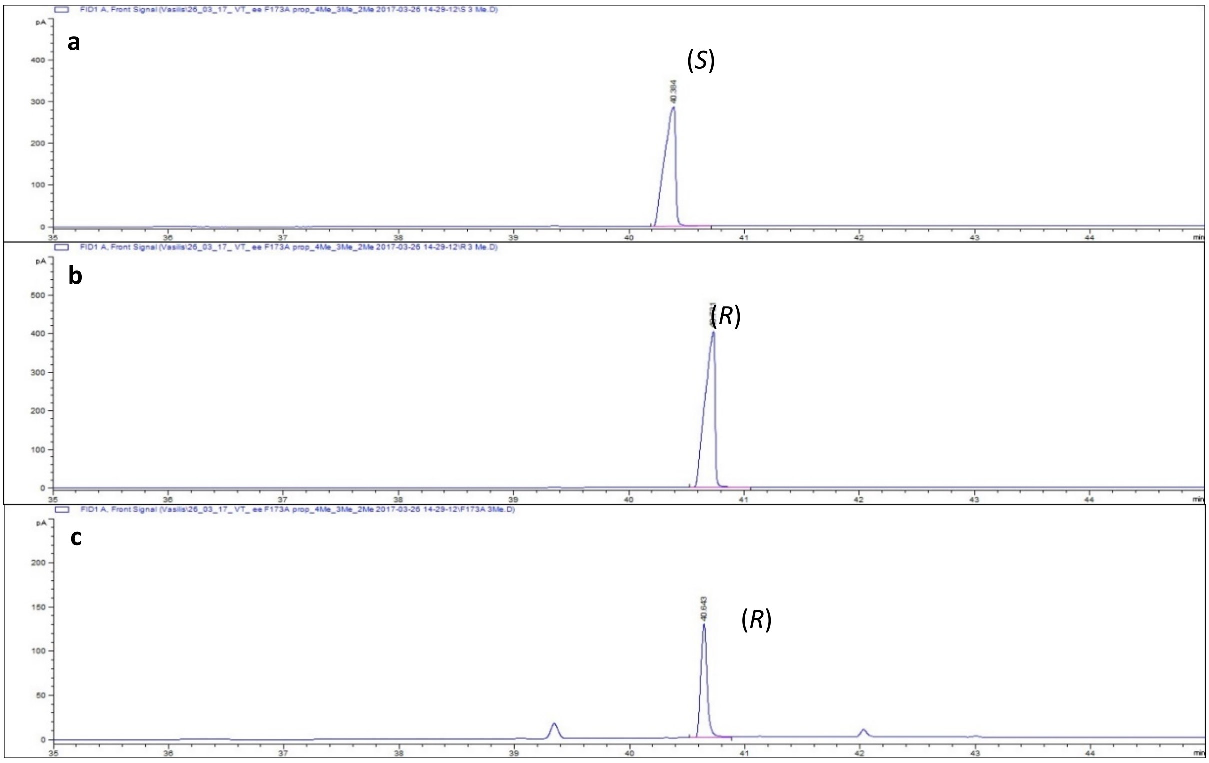


**Supplementary Figure 17**. **GC-FID chromatograms for the determination of the absolute configuration of 20b.** (a) reference *S*-configured amine synthetized by the *S*-selective Cv-ωTA. (b) reference *R*-configured amine synthetized by the *R*-selective As-ωTA. (c) Biocatalytic reaction catalysed by LE-AmDH-v1. The areas obtained from the reaction with LE-AmDH-v1 were 487 for the *R*- and 0 for the *S*-configured amines.

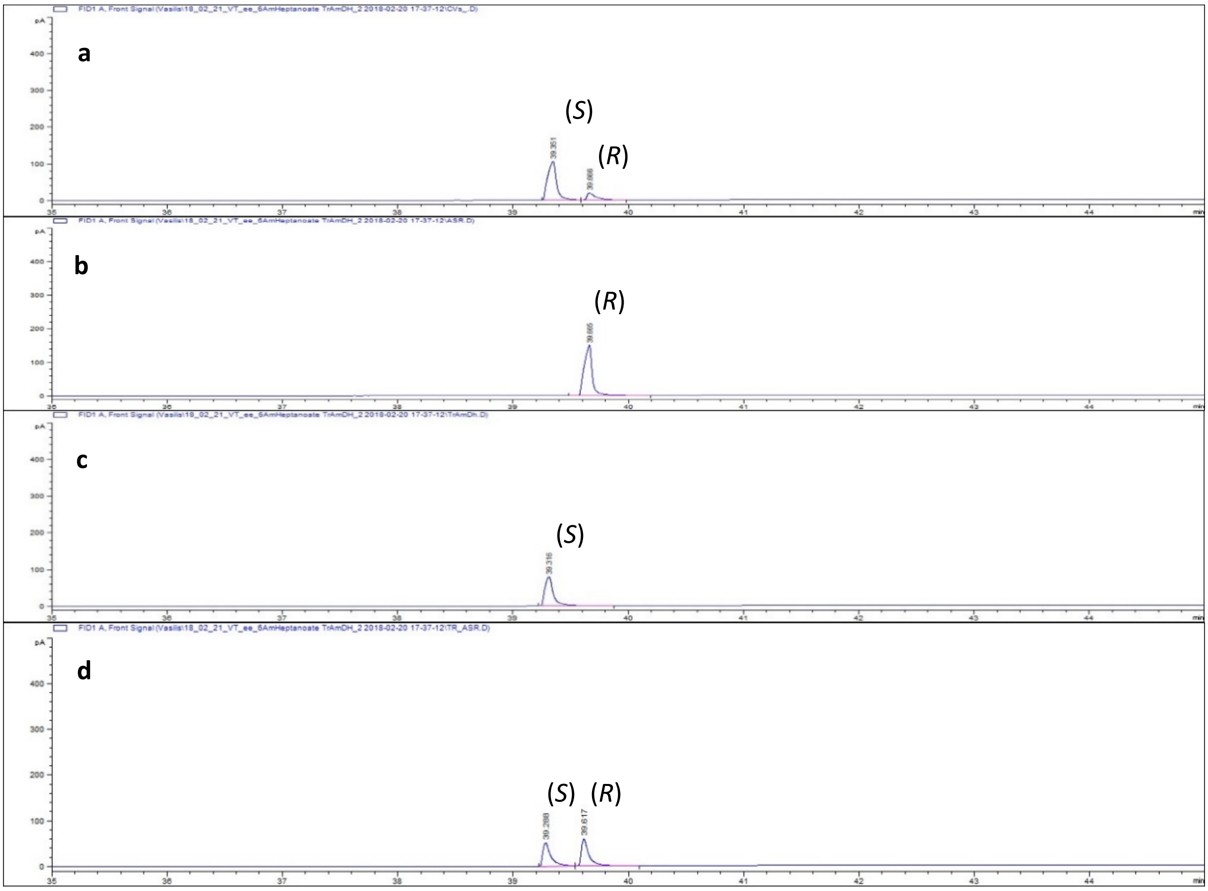


**Supplementary Figure 18**. **GC-FID chromatograms for the determination of the absolute configuration of 22b.** 22b is the methyl ester of **3b** (a) reference *S*-configured amine synthetized by the *S*-selective Cv-ωTA. (b) reference *R*-configured amine synthetized by the *R*-selective As-ωTA. (c) Biocatalytic reaction catalysed by LE-AmDH-v1. (d) Co-injection of the reactions catalysed by LE-amDH-v1 and As-ωTA. The areas obtained from the reaction with LE-AmDH-v1 were 0 for the *R*- and 411 for the *S*-configured amines.

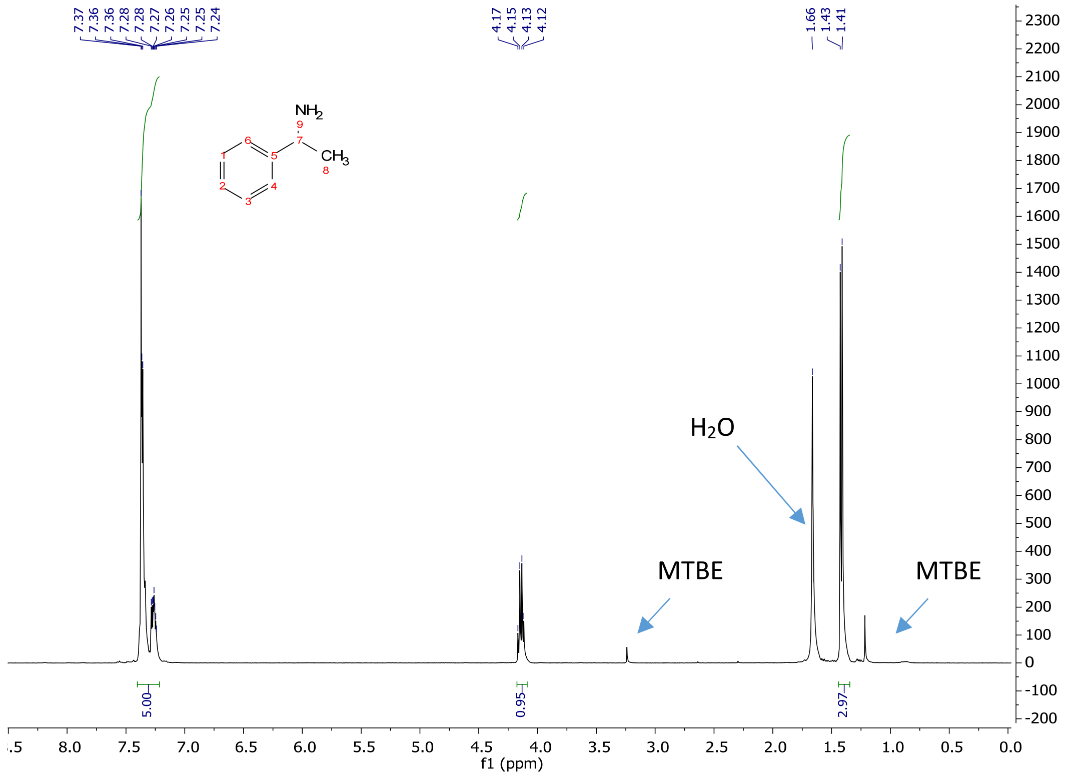


**Supplementary Figure 19. $^1$H-NMR spectrum of isolated 8b.** The spectrum was recorded in $CDCl_3$ after isolation from the biocatalytic reaction catalysed by LE-AmDH-v1 on preparative scale, on a Bruker Avance 400 MHz NMR spectrometer.

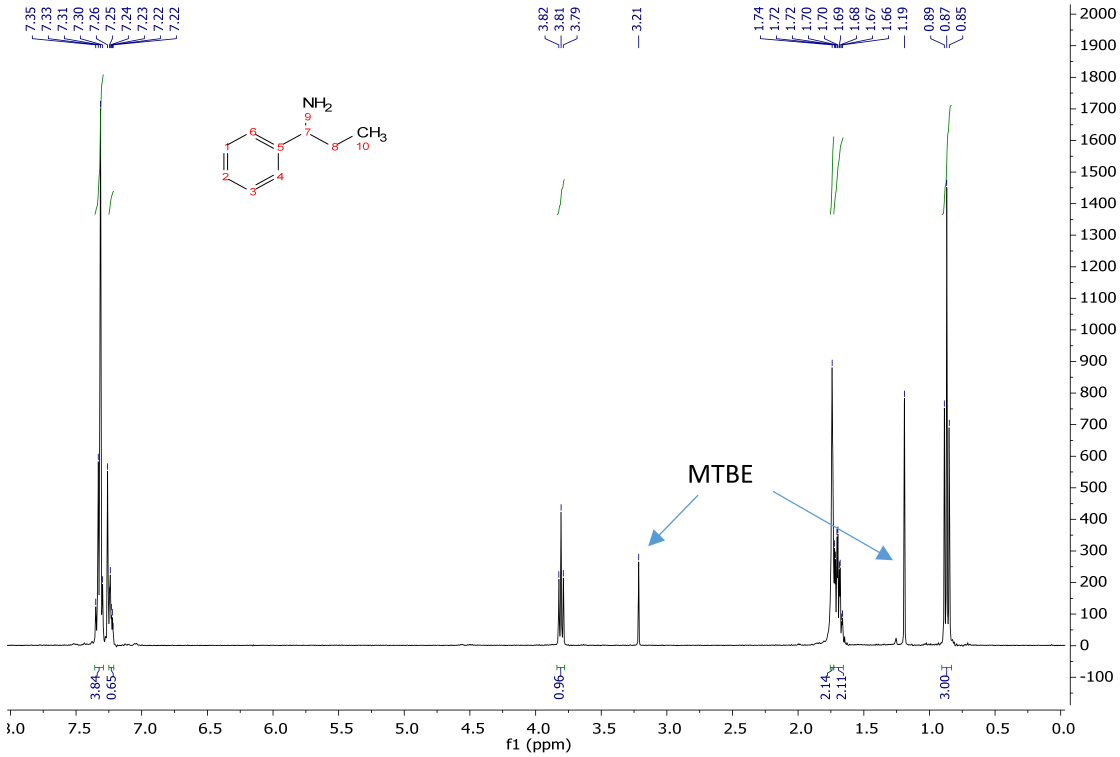


**Supplementary Figure 20**. **$^1$H-NMR spectrum of isolated 9b.** The spectrum was recorded in $CDCl_3$ after isolation from the biocatalytic reaction catalysed by LE-AmDH-v1 on preparative scale, on a Bruker Avance 400 MHz NMR spectrometer.

## Supplementary Tables

**Supplementary Table 1**. Retention times for the obtained products measured by GC-FID or RP-HPLC.

| Substrate | retention time [min] | Primary Amine | retention time [min] | method |
|---|---|---|---|---|
| **2a** | 16.1 | **2b** | 5.0 | RP HPLC-A |
| **3a** | 35.3 | **3b** | 9.6 | RP HPLC-A |
| **4a** | 5.22 | **4b** | 4.05 | DB1701-30m-B |
| **8a** | 12.31 | **8b** | 11.49 | DB1701-30m-B |
| **9a** | 13.42 | **9b** | 12.69 | DB1701-30m-B |
| **10a** | 14.30 | **10b** | 13.73 | DB1701-30m-B |
| **11a** | 15.21 | **11b** | 14.66 | DB1701-30m-B |
| **12a** | 15.75 | **12b** | 15.19 | DB1701-30m-B |
| **13a** | 15.49 | **13b** | 15.38 | DB1701-30m-B |
| **14a** | 8.85 | **14b** | 6.41 | DB1701-30m-B |
| **15a** | 3.22 | **15b** | 2.73 | DB1701-30m-B |
| **16a** | 4.17 | **16b** | 3.56 | DB1701-30m-B |
| **17a** | 12.22 | **17b** | 11.81 | DB1701-30m-B |
| **18a** | 12.10 | **18b** | 11.89 | DB1701-30m-B |
| **19a** | 11.42 | **19b** | 11.56 | DB1701-30m-B |
| **20a** | 13.59 | **20b** | 12.86 | DB1701-30m-B |
| **21a** | 10.43 | **21b** | 11.08 | DB1701-30m-B |

**Supplementary Table 2**. GC retention times of the derivatised enantiomerically pure amines measured byGC-FID.

| number | retention time [min] | number | retention time [min] | method |
|---|---|---|---|---|
| (*S*)-**4b** | 15.46 | (*R*)-**4b** | 15.69 | DEX-CB-A |
| (*S*)-**8b** | 38.0 | (*R*)-**8b** | 38.9 | DEX-CB-A |
| (*S*)-**9b** | 39.1 | (*R*)-**9b** | 39.5 | DEX-CB-A |
| (*S*)-**10b** | 41.1 | (*R*)-**10b** | 41.2 | DEX-CB-A |
| (*S*)-**11b** | 41.62 | (*R*)-**11b** | 41.58 | DEX-CB-A |
| (*S*)-**12b** | 32.07 | (*R*)-**12b** | 32.53 | DEX-CB-A |
| (*S*)-**13b** | 47.7 | (*R*)-**13b** | 48.6 | DEX-CB-A |
| (*S*)-**15b** | 8.7 | (*R*)-**15b** | 9.2 | DEX-CB-A |
| (*S*)-**16b** | 10.0 | (*R*)-**16b** | 10.6 | DEX-CB-A |
| (*S*)-**17b** | 38.8 | (*R*)-**17b** | 39.3 | DEX-CB-A |
| (*S*)-**18b** | 37.62 | (*R*)-**18b** | 38.58 | DEX-CB-A |
| (*S*)-**19b** | 29.2 | (*R*)-**19b** | 29.8 | DEX-CB-A |
| (*S*)-**20b** | 40.38 | (*R*)-**20b** | 40.73 | DEX-CB-A |
| (*S*)-**22b** | 39.35 | (*R*)-**22b** | 39.36 | DEX-CB-A |

**Supplementary Table 3.** Results for the reductive amination of **4a**-**6a**.

| Substrate | *LE-AmDH* | Recovered substrate | Amine | Imine intermediate | Alcohol | Amine |
|---|---|---|---|---|---|---|
| **10 mM** | **90 µM** | **(%)** | | **Conv. (%)**[a] | | ***ee* (%)** |
| 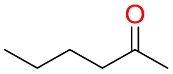 **4a** | ***LE-AmDH-v1*** | 22 | 78 | n.d. | n.d. | >99% *(R)* |
| | ***LE-AmDH-v2*** | 100 | n.d. | n.d. | n.d. | - |
| | ***LE-AmDH-v3*** | 100 | n.d. | n.d. | n.d. | - |
| | ***LE-AmDH-v4*** | 100 | n.d. | n.d. | n.d. | - |
| | ***LE-AmDH-v5*** | 100 | n.d. | n.d. | n.d. | - |
| | ***LE-AmDH-v6*** | 100 | n.d. | n.d. | n.d. | - |
| | ***LE-AmDH-v7*** | 100 | n.d. | n.d. | n.d. | - |
| | ***LE-AmDH-v8*** | 100 | n.d. | n.d. | n.d. | - |
| | ***LE-AmDH-v9*** | 100 | n.d. | n.d. | n.d. | - |
| | ***LE-AmDH-v10*** | 100 | n.d. | n.d. | n.d. | - |
| | ***LE-AmDH-v11*** | 100 | n.d. | n.d. | n.d. | - |
| | ***LE-AmDH-v12*** | 37 | 64 | n.d. | n.d. | 83% *(R)* |
| | ***LE-AmDH-v13*** | 100 | n.d. | n.d. | n.d. | - |
| | ***LE-AmDH-v14*** | 100 | n.d. | n.d. | n.d. | - |
| 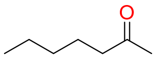 **5a** | ***LE-AmDH-v1*** | 90 | 10 | n.d. | n.d. | >99% *(R)* |
| | ***LE-AmDH-v2*** | 100 | n.d. | n.d. | n.d. | - |
| | ***LE-AmDH-v3*** | 100 | n.d. | n.d. | n.d. | - |
| | ***LE-AmDH-v4*** | 100 | n.d. | n.d. | n.d. | - |
| | ***LE-AmDH-v5*** | 100 | n.d. | n.d. | n.d. | - |
| | ***LE-AmDH-v6*** | 100 | n.d. | n.d. | n.d. | - |
| | ***LE-AmDH-v7*** | 100 | n.d. | n.d. | n.d. | - |
| | ***LE-AmDH-v8*** | 100 | n.d. | n.d. | n.d. | - |
| | ***LE-AmDH-v9*** | 100 | n.d. | n.d. | n.d. | - |
| | ***LE-AmDH-v10*** | 100 | n.d. | n.d. | n.d. | - |
| | ***LE-AmDH-v11*** | 98 | 2 | n.d. | n.d. | N.m. |
| | ***LE-AmDH-v12*** | 89 | 11 | n.d. | n.d. | >99% *(R)* |
| | ***LE-AmDH-v13*** | 100 | n.d. | n.d. | n.d. | - |
| | ***LE-AmDH-v14*** | 100 | n.d. | n.d. | n.d. | - |
| 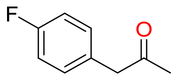 **6a** | ***LE-AmDH-v1*** | 100 | n.d. | n.d. | n.d. | - |
| | ***LE-AmDH-v2*** | 100 | n.d. | n.d. | n.d. | - |
| | ***LE-AmDH-v3*** | 100 | n.d. | n.d. | n.d. | - |
| | ***LE-AmDH-v4*** | 100 | n.d. | n.d. | n.d. | - |
| | ***LE-AmDH-v5*** | 100 | n.d. | n.d. | n.d. | - |
| | ***LE-AmDH-v6*** | 100 | n.d. | n.d. | n.d. | - |
| | ***LE-AmDH-v7*** | 100 | n.d. | n.d. | n.d. | - |
| | ***LE-AmDH-v8*** | 100 | n.d. | n.d. | n.d. | - |
| | ***LE-AmDH-v9*** | 100 | n.d. | n.d. | n.d. | - |
| | ***LE-AmDH-v10*** | 100 | n.d. | n.d. | n.d. | - |
| | ***LE-AmDH-v11*** | 100 | n.d. | n.d. | n.d. | - |
| | ***LE-AmDH-v12*** | 100 | n.d. | n.d. | n.d. | - |
| | ***LE-AmDH-v13*** | 100 | n.d. | n.d. | n.d. | - |
| | ***LE-AmDH-v14*** | 100 | n.d. | n.d. | n.d. | - |

[a] Conversions were determined using a 7890A GC system (Agilent Technologies), equipped with FID detector using $H_2$ as carrier gas with a DB-1701 column from Agilent (30 m, 250 µm, 0.25 µm)

Biocatalytic reactions were performed as described in the main paper at 30 °C for 48 h. Experimental conditions: 0.5 mL final volume in Eppendorf tubes, buffer = $HCOONH_4/NH_3$ 2 M, pH 9.0; 170 rpm on orbital shaker, [substrate] = 10 mM, [$NAD^+$] = 1 mM, [enzyme] = 90 µM, [Cb-FDH] = 19 µM.

**Supplementary Table 4.** Results for the reductive amination of **7a**-**9a**.

| Substrate | *LE-AmDH* | Recovered substrate | Amine | Imine intermediate | Alcohol | Amine |
|---|---|---|---|---|---|---|
| **10 mM** | **90 µM** | **(%)** | **Conv. (%)[a]** | ***ee* (%)** | | |
| 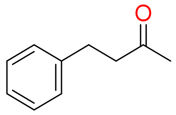 **7a** | ***LE-AmDH-v1*** | 100 | n.d. | n.d. | n.d. | - |
| | ***LE-AmDH-v2*** | 100 | n.d. | n.d. | n.d. | - |
| | ***LE-AmDH-v3*** | 100 | n.d. | n.d. | n.d. | - |
| | ***LE-AmDH-v4*** | 100 | n.d. | n.d. | n.d. | - |
| | ***LE-AmDH-v5*** | 100 | n.d. | n.d. | n.d. | - |
| | ***LE-AmDH-v6*** | 100 | n.d. | n.d. | n.d. | - |
| | ***LE-AmDH-v7*** | 100 | n.d. | n.d. | n.d. | - |
| | ***LE-AmDH-v8*** | 100 | n.d. | n.d. | n.d. | - |
| | ***LE-AmDH-v9*** | 100 | n.d. | n.d. | n.d. | - |
| | ***LE-AmDH-v10*** | 100 | n.d. | n.d. | n.d. | - |
| | ***LE-AmDH-v11*** | >99 | <1 | n.d. | n.d. | - |
| | ***LE-AmDH-v12*** | 99 | 1 | n.d. | n.d. | - |
| | ***LE-AmDH-v13*** | 100 | n.d. | n.d. | n.d. | - |
| | ***LE-AmDH-v14*** | 100 | n.d. | n.d. | n.d. | - |
| 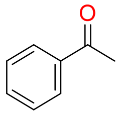 **8a** | ***LE-AmDH-v1*** | 2 | 98 | n.d. | n.d. | >99% *(R)* |
| | ***LE-AmDH-v2*** | 70 | 30 | n.d. | n.d. | - |
| | ***LE-AmDH-v3*** | 90 | 10 | n.d. | n.d. | - |
| | ***LE-AmDH-v4*** | 100 | n.d. | n.d. | n.d. | - |
| | ***LE-AmDH-v5*** | 88 | 12 | n.d. | n.d. | - |
| | ***LE-AmDH-v6*** | 77 | 23 | n.d. | n.d. | - |
| | ***LE-AmDH-v7*** | 94[c] | 6[c] | n.d. | n.d. | - |
| | ***LE-AmDH-v8*** | >99 | <1 | n.d. | n.d. | - |
| | ***LE-AmDH-v9*** | 100 | n.d. | n.d. | n.d. | - |
| | ***LE-AmDH-v10*** | 100 | n.d. | n.d. | n.d. | - |
| | ***LE-AmDH-v11*** | 26 | 74 | n.d. | n.d. | >99% *(R)* |
| | ***LE-AmDH-v12*** | 6 | 9 | n.d. | n.d. | >99% *(R)* |
| | ***LE-AmDH-v13*** | 62 | 38 | n.d. | n.d. | >99% *(R)* |
| | ***LE-AmDH-v14*** | 100 | n.d. | n.d. | n.d. | - |
| 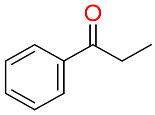 **9a** | ***LE-AmDH-v1*** | 11 | 89 | n.d. | n.d. | >99% *(R)* |
| | ***LE-AmDH-v2*** | 73 | 27 | n.d. | n.d. | - |
| | ***LE-AmDH-v3*** | 92 | 8 | n.d. | n.d. | - |
| | ***LE-AmDH-v4*** | 100 | n.d. | n.d. | n.d. | - |
| | ***LE-AmDH-v5*** | 86 | 14 | n.d. | n.d. | - |
| | ***LE-AmDH-v6*** | 82 | 18 | n.d. | n.d. | - |
| | ***LE-AmDH-v7*** | 81 | 19 | n.d. | n.d. | >99% *(R)* |
| | ***LE-AmDH-v8*** | 100 | n.d. | n.d. | n.d. | - |
| | ***LE-AmDH-v9*** | 100 | n.d. | n.d. | n.d. | - |
| | ***LE-AmDH-v10*** | 100 | n.d. | n.d. | n.d. | - |
| | ***LE-AmDH-v11*** | 35 | 65 | n.d. | n.d. | >99% *(R)* |
| | ***LE-AmDH-v12*** | 22 | 78 | n.d. | n.d. | >99% *(R)* |
| | ***LE-AmDH-v13*** | 59 | 41 | n.d. | n.d. | >99% *(R)* |
| | ***LE-AmDH-v14*** | 100 | n.d. | n.d. | n.d. | - |

[a] Conversions were determined using a 7890A GC system (Agilent Technologies), equipped with FID detector using $H_2$ as carrier gas with a DB-1701 column from Agilent (30 m, 250 µm, 0.25 µm)
Biocatalytic reactions were performed as described in the main paper at 30 °C for 48 h. Experimental conditions: 0.5 mL final volume in Eppendorf tubes, buffer = HCOONH4/NH3 2 M, pH 9.0; 170 rpm on orbital shaker, [substrate] = 10 mM, [NAD+] = 1 mM, [enzyme] = 90 µM, [Cb-FDH] = 19 µM.

**Supplementary Table 5**. Results for the reductive amination of **10a**-**12a**.

| Substrate | *LE-AmDH* | Recovered substrate | Amine | Imine intermediate | Alcohol | Amine |
|---|---|---|---|---|---|---|
| **10 mM** | **90 µM** | **(%)** | **Conv. (%)[a]** | ***ee* (%)** | | |
| 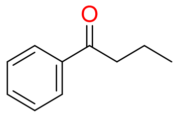 **10a** | ***LE-AmDH-v1*** | 59 | 41 | n.d. | n.d. | >99% *(R)* |
| | ***LE-AmDH-v2*** | 81 | 19 | n.d. | n.d. | - |
| | ***LE-AmDH-v3*** | 93 | 7 | n.d. | n.d. | - |
| | ***LE-AmDH-v4*** | n.d. | n.d. | n.d. | n.d. | - |
| | ***LE-AmDH-v5*** | 89 | 11 | n.d. | n.d. | - |
| | ***LE-AmDH-v6*** | 50 | 6 | n.d. | n.d. | - |
| | ***LE-AmDH-v9*** | 100 | n.d. | n.d. | n.d. | - |
| | ***LE-AmDH-v10*** | 100 | n.d. | n.d. | n.d. | - |
| | ***LE-AmDH-v11*** | 59 | 41 | n.d. | n.d. | >99% *(R)* |
| | ***LE-AmDH-v12*** | 52 | 48 | n.d. | n.d. | >99% *(R)* |
| | ***LE-AmDH-v13*** | 62 | 38 | n.d. | n.d. | >99% *(R)* |
| | ***LE-AmDH-v14*** | 100 | n.d. | n.d. | n.d. | - |
| 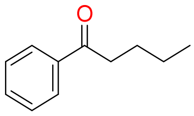 **11a** | ***LE-AmDH-v1*** | 80 | 20 | n.d. | n.d. | *(R)* [b] |
| | ***LE-AmDH-v2*** | 97 | 3 | n.d. | n.d. | - |
| | ***LE-AmDH-v3*** | 100 | n.d. | n.d. | n.d. | - |
| | ***LE-AmDH-v4*** | 100 | n.d. | n.d. | n.d. | - |
| | ***LE-AmDH-v5*** | 100 | n.d. | n.d. | n.d. | - |
| | ***LE-AmDH-v6*** | 100 | n.d. | n.d. | n.d. | - |
| | ***LE-AmDH-v9*** | 100 | n.d. | n.d. | n.d. | - |
| | ***LE-AmDH-v10*** | 100 | n.d. | n.d. | n.d. | - |
| | ***LE-AmDH-v11*** | 81 | 19 | n.d. | n.d. | *(R)* [b] |
| | ***LE-AmDH-v12*** | 100 | n.d. | n.d. | n.d. | - |
| | ***LE-AmDH-v13*** | 100 | n.d. | n.d. | n.d. | - |
| | ***LE-AmDH-v14*** | 100 | n.d. | n.d. | n.d. | - |
| 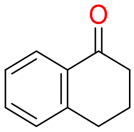 **12a** | ***LE-AmDH-v1*** | 39 | 61 | n.d. | n.d. | >99% *(R)* |
| | ***LE-AmDH-v2*** | 100 | n.d. | n.d. | n.d. | - |
| | ***LE-AmDH-v3*** | 100 | n.d. | n.d. | n.d. | - |
| | ***LE-AmDH-v4*** | 100 | n.d. | n.d. | n.d. | - |
| | ***LE-AmDH-v5*** | 100 | n.d. | n.d. | n.d. | - |
| | ***LE-AmDH-v6*** | 100 | n.d. | n.d. | n.d. | - |
| | ***LE-AmDH-v7*** | >99 | <1 | n.d. | n.d. | - |
| | ***LE-AmDH-v8*** | 100 | n.d. | n.d. | n.d. | - |
| | ***LE-AmDH-v9*** | 100 | n.d. | n.d. | n.d. | - |
| | ***LE-AmDH-v10*** | 100 | n.d. | n.d. | n.d. | - |
| | ***LE-AmDH-v11*** | 92 | 8 | n.d. | n.d. | >99% *(R)* |
| | ***LE-AmDH-v12*** | 66 | 34 | n.d. | n.d. | >99% *(R)* |
| | ***LE-AmDH-v13*** | 100 | n.d. | n.d. | n.d. | - |
| | ***LE-AmDH-v14*** | 97 | 3 | n.d. | n.d. | >99% *(R)* |

[a] Conversions were determined using a 7890A GC system (Agilent Technologies), equipped with FID detector using $H_2$ as carrier gas with a DB-1701 column from Agilent (30 m, 250 µm, 0.25 µm)
[b] Unable to determine the *ee* due to partially overlap of the enantiomers with the method used
Biocatalytic reactions were performed as described in the main paper at 30 °C for 48 h. Experimental conditions: 0.5 mL final volume in Eppendorf tubes, buffer = $HCOONH_4/NH_3$ 2 M, pH 9.0; 170 rpm on orbital shaker, [substrate] = 10 mM, [$NAD^+$] = 1 mM, [enzyme] = 90 µM, [Cb-FDH] = 19 µM.

**Supplementary Table 6** Results for the reductive amination of **13a**-**14a**.

| Substrate | *LE-AmDH* | Recovered substrate | Amine | Imine intermediate | Alcohol | Amine |
|---|---|---|---|---|---|---|
| **10 mM** | **90 µM** | **(%)** | **Conv. (%)[a]** | ***ee* (%)** | | |
| 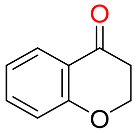 **13a** | ***LE-AmDH-v1*** | 30 | 70 | n.d. | n.d. | >99% *(R)* |
| | ***LE-AmDH-v2*** | 96 | 4 | n.d. | n.d. | -. |
| | ***LE-AmDH-v3*** | 100 | n.d. | n.d. | n.d. | - |
| | ***LE-AmDH-v4*** | 100 | n.d. | n.d. | n.d. | - |
| | ***LE-AmDH-v5*** | 100 | n.d. | n.d. | n.d. | - |
| | ***LE-AmDH-v6*** | 100 | n.d. | n.d. | n.d. | - |
| | ***LE-AmDH-v7*** | 100 | n.d. | n.d. | n.d. | - |
| | ***LE-AmDH-v8*** | 100 | n.d. | n.d. | n.d. | - |
| | ***LE-AmDH-v9*** | 100 | n.d. | n.d. | n.d. | - |
| | ***LE-AmDH-v10*** | 100 | n.d. | n.d. | n.d. | - |
| | ***LE-AmDH-v11*** | 64 | 36 | n.d. | n.d. | >99% *(R)* |
| | ***LE-AmDH-v12*** | 64 | 36 | n.d. | n.d. | >99% *(R)* |
| | ***LE-AmDH-v13*** | 85 | 15 | n.d. | n.d. | >99% *(R)* |
| | ***LE-AmDH-v14*** | 100 | n.d. | n.d. | n.d. | - |
| 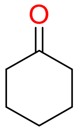 **14a** | ***LE-AmDH-v1*** | 49 | 51 | n.d. | n.d. | N.a. |
| | ***LE-AmDH-v3*** | 100 | n.d. | n.d. | n.d. | N.a. |
| | ***LE-AmDH-v4*** | 100 | n.d. | n.d. | n.d. | N.a. |
| | ***LE-AmDH-v5*** | 100 | n.d. | n.d. | n.d. | N.a. |
| | ***LE-AmDH-v6*** | 100 | n.d. | n.d. | n.d. | N.a. |
| | ***LE-AmDH-v7*** | 100 | n.d. | n.d. | n.d. | N.a. |
| | ***LE-AmDH-v8*** | 100 | n.d. | n.d. | n.d. | N.a. |
| | ***LE-AmDH-v9*** | 100 | n.d. | n.d. | n.d. | N.a. |
| | ***LE-AmDH-v10*** | 100 | n.d. | n.d. | n.d. | N.a. |
| | ***LE-AmDH-v11*** | 96 | 4 | n.d. | n.d. | N.a. |
| | ***LE-AmDH-v12*** | 70 | 30 | n.d. | n.d. | N.a. |
| | ***LE-AmDH-v13*** | 100 | n.d. | n.d. | n.d. | N.a. |
| | ***LE-AmDH-v14*** | 100 | n.d. | n.d. | n.d. | N.a. |

[a] Conversions were determined using a 7890A GC system (Agilent Technologies), equipped with FID detector using $H_2$ as carrier gas with a DB-1701 column from Agilent (30 m, 250 µm, 0.25 µm)

Biocatalytic reactions were performed as described in the main paper at 30 °C for 48 h. Experimental conditions: 0.5 mL final volume in Eppendorf tubes, buffer = $HCOONH_4/NH_3$ 2 M, pH 9.0; 170 rpm on orbital shaker, [substrate] = 10 mM, [$NAD^+$] = 1 mM, [enzyme] = 90 µM, [Cb-FDH] = 19 µM. N.a: not applicable

**Supplementary Table 7**. Results for the reductive amination of **15a-16a**.

| **Substrate** | ***LE-AmDH*** | **Recovered substrate** | **Amine** | **Imine intermediate** | **Alcohol** | **Amine** |
|---|---|---|---|---|---|---|
| **10 mM** | **90 µM** | **(%)** | **Conv. (%)[a]** | ***ee* (%)** | | |
| 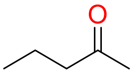 **15a** | ***LE-AmDH-v1*** | **63** | **26** | **n.d.** | **n.d.** | 89% *(R)* |
| | ***LE-AmDH-v2*** | 100 | n.d. | n.d. | n.d. | - |
| | ***LE-AmDH-v3*** | 100 | n.d. | n.d. | n.d. | - |
| | ***LE-AmDH-v4*** | 100 | n.d. | n.d. | n.d. | - |
| | ***LE-AmDH-v5*** | 100 | n.d. | n.d. | n.d. | - |
| | ***LE-AmDH-v6*** | 100 | n.d. | n.d. | n.d. | - |
| | ***LE-AmDH-v7*** | 100 | n.d. | n.d. | n.d. | - |
| | ***LE-AmDH-v8*** | 100 | n.d. | n.d. | n.d. | - |
| | ***LE-AmDH-v9*** | 100 | n.d. | n.d. | n.d. | - |
| | ***LE-AmDH-v10*** | 100 | n.d. | n.d. | n.d. | - |
| | ***LE-AmDH-v11*** | 24 | 2 | n.d. | n.d. | - |
| | ***LE-AmDH-v12*** | 58 | 42 | n.d. | n.d. | 77% *(R)* |
| | ***LE-AmDH-v13*** | 100 | n.d. | n.d. | n.d. | - |
| | ***LE-AmDH-v14*** | 100 | n.d. | n.d. | n.d. | - |
| 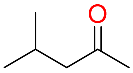 **16a** | ***LE-AmDH-v1*** | **42** | **58** | **n.d.** | **n.d.** | 97% *(R)* |
| | ***LE-AmDH-v2*** | 100 | n.d. | n.d. | n.d. | - |
| | ***LE-AmDH-v3*** | 100 | n.d. | n.d. | n.d. | - |
| | ***LE-AmDH-v4*** | 100 | n.d. | n.d. | n.d. | - |
| | ***LE-AmDH-v5*** | 100 | n.d. | n.d. | n.d. | - |
| | ***LE-AmDH-v6*** | 100 | n.d. | n.d. | n.d. | - |
| | ***LE-AmDH-v7*** | 100 | n.d. | n.d. | n.d. | - |
| | ***LE-AmDH-v8*** | 100 | n.d. | n.d. | n.d. | - |
| | ***LE-AmDH-v9*** | 100 | n.d. | n.d. | n.d. | - |
| | ***LE-AmDH-v10*** | 100 | n.d. | n.d. | n.d. | - |
| | ***LE-AmDH-v11*** | 95 | 5 | n.d. | n.d. | n.m. |
| | ***LE-AmDH-v12*** | 62 | 38 | n.d. | n.d. | >99% *(R)* |
| | ***LE-AmDH-v13*** | 100 | n.d. | n.d. | n.d. | - |
| | ***LE-AmDH-v14*** | 100 | n.d. | n.d. | n.d. | - |

[a] Conversions were determined using a 7890A GC system (Agilent Technologies), equipped with FID detector using $H_2$ as carrier gas with a DB-1701 column from Agilent (30 m, 250 µm, 0.25 µm)

Biocatalytic reactions were performed as described in the main paper at 30 °C for 48 h. Experimental conditions: 0.5 mL final volume in Eppendorf tubes, buffer = $HCOONH_4/NH_3$ 2 M, pH 9.0; 170 rpm on orbital shaker, [substrate] = 10 mM, [$NAD^+$] = 1 mM, [enzyme] = 90 µM, [Cb-FDH] = 19 µM. N.d: not determined

**Supplementary Table 8.** Progress for the reductive amination of **8a** over time using LE-AmDH-v1 at different temperatures.

| **Time (h)** | **Conversion (%)** | | | | |
|---|---|---|---|---|---|
| | 20 °C | 30 °C | 40 °C | 50 °C | 60 °C |
| **1** | 9.6 | 16.3 | 23.7 | 32.1 | 43.6 |
| **2** | 15.3 | 27.7 | 42.4 | 53.0 | 65.2 |
| **4** | 28.3 | 45.7 | 67.1 | 79.7 | 89.0 |
| **8** | 46.3 | 63.9 | 83.0 | 94.1 | 91.2 |
| **16** | 71.3 | 90.9 | 97.3 | 98.6 | 90.4 |
| **20** | 78.4 | 94.7 | 98.4 | 98.8 | 89.2 |
| **24** | 81.6 | 96.5 | 98.8 | 99.3 | 95.4 |
| **32** | 88.1 | 97.9 | 99.3 | 99.5 | 98.6 |
| **48** | 94.6 | 98.6 | 99.6 | 100 | 95.7 |

The study was carried out from 20 up to 60 °C as described in the main paper and reactions were stopped at different time points (1, 2, 4, 8, 16, 20, 24, 32 and 48 h). The results are summarized in **Figure 3a** in the main paper.Experimental conditions: 0.5 mL final volume in Eppendorf tubes, buffer = $HCOONH_4/NH_3$ 2 M, pH 9.0; 170 rpm on orbital shaker, [**8a**] = 10 mM, [$NAD^+$] = 1 mM, [LE-AmDH-v1] = 90 µM, [Cb-FDH] = 19 µM.

**Supplementary Table 9.** Progress for the reductive amination of **8a** over time at different pH values of the $HCOONH_4/NH_3$ buffer.

| **Time (h)** | **Conversion (%)** | | | | |
|---|---|---|---|---|---|
| | pH 7.0 | pH 8.0 | pH 8.5 | pH 9.0 | pH 9.5 |
| **1** | 7.1 | 10.4 | 16.7 | 29.1 | 28.9 |
| **2** | 16.9 | 23.8 | 33.0 | 59.4 | 56.4 |
| **4** | 34.7 | 45.2 | 59.6 | 83.4 | 82.5 |
| **8** | 60.9 | 71.3 | 82.6 | 95.4 | 95.5 |
| **24** | 86.9 | 94.8 | 97.2 | 99.3 | 99.2 |

The influence of the pH value (7.0, 8.0, 8.5, 9.0 and 9.5) on the buffer ($HCOONH_4/NH_3$ 2 M) was investigated for the reductive amination of **8a** using LE-AmDH-v1 as described in the main paper. The results are summarized in **Figure 3b** in the main paper. Experimental conditions: 0.5 mL final volume in Eppendorf tubes, buffer = $HCOONH_4/NH_3$ 2 M; 170 rpm on orbital shaker, [**8a**] = 10 mM, [$NAD^+$] = 1 mM, [LE-AmDH-v1] = 90 µM, [Cb-FDH] = 19 µM.

**Supplementary Table 10.** Conversions obtained by increasing the concentration of selected substrates.

| | Conversion (%) | | | | | | | | | |
|---|---|---|---|---|---|---|---|---|---|---|
| **Substrate** | **10 mM** | | **30 mM** | | **50 mM** | | **75 mM** | | **100 mM** | |
| | **50 °C** | **30 °C** | **50 °C** | **30 °C** | **50 °C** | **30 °C** | **50 °C** | **30°C** | **50 °C** | **30°C** |
| **8a** | >99 | 98 | >99 | 96 | 98 | 93 | 86 | 79 | 84 | 35 |
| **13a** | 82 | 70 | 74 | 67 | 55 | 57 | 28 | 42 | 20 | 33 |
| **9a** | 98 | 89 | 98 | 87 | 95 | 70 | 92 | 53 | 89 | 34 |
| **17a** | 99 | 90 | 96 | 82 | 92 | 74 | 28 | 55 | 25 | 40 |
| **18a** | 99 | 92 | 97 | 81 | 96 | 67 | 68 | 52 | 58 | 28 |
| **19a** | >99 | 96 | 99 | 92 | 98 | 78 | 93 | 32 | 47 | 24 |
| **21a** | >99 | >99 | >99 | >99 | >99 | >99 | >99 | >99 | >99 | >99 |

The influence of the substrate concentration for 8a, 9a, 12a, 13a, 17a-19a and 21a (10 mM to 100 mM) was investigated at 30 °C and 50 °C using LE-AmDH-v1. The conversions and productivities are summarized in Figure 3c-i in the main paper. Experimental conditions: 0.5 mL final volume in Eppendorf tubes, buffer = HCOONH4/NH3 2 M pH 9.0; 170 rpm on orbital shaker, [substrate] = 10 mM up to 100 mM, [NAD+] = 1 mM, [LE-AmDH-v1] = 90 µM, [Cb-FDH] = 19 µM.

**Supplementary Table 11.** Calculated productivity (mM) by increasing the concentration of selected substrates.

| | productivity (mM) | | | | | | | | | |
|---|---|---|---|---|---|---|---|---|---|---|
| **Substrate** | **10 mM** | | **30 mM** | | **50 mM** | | **75 mM** | | **100 mM** | |
| | **50 °C** | **30°C** | **50 °C** | **30°C** | **50 °C** | **30°C** | **50 °C** | **30 °C** | **50 °C** | **30 °C** |
| **8a** | 10 | 9.8 | 29.9 | 28.8 | 49 | 47 | 64.5 | 59.3 | 84 | 35 |
| **13a** | 9.8 | 8.5 | 27 | 23.7 | 20 | 33.5 | 22 | 38.3 | 20 | 37 |
| **9a** | 9.8 | 8.9 | 29.4 | 26.1 | 47.5 | 35 | 69 | 39.8 | 89 | 34 |
| **17a** | 9.9 | 9 | 28.8 | 24.6 | 46 | 37 | 21 | 41.3 | 25 | 40 |
| **18a** | 9.9 | 9.2 | 29.1 | 24.3 | 48 | 33.5 | 51 | 39 | 58 | 28 |
| **19a** | 10 | 9.6 | 29.7 | 27.6 | 49 | 39 | 69.6 | 24 | 47 | 24 |
| **21a** | 10 | 10 | 30 | 30 | 50 | 50 | 75 | 75 | >99 | >99 |

**Supplementary Table 12**. **Homology models of the wild-type LysEDH**. The full amino acid sequence was explored. The multimeric state, the selected templates, the excluded templates, and the number of models generated per run are shown. The template that was selected as main contributor to the hybrid model is demarked in bold font. Moreover, the accuracy of the generated models is reported by the use of Z-scores[6,7]. The overall Z-scores for all models have been calculated as the weighted averages of the individual Z-scores (Dihedrals, Packing 1D, and Packing 3D) using the **Supplementary equation 1**.

| Model | Multimeric state | Templates | Excluded templates | Cofactor | Ligand | Number of models | Overall Z-score (Quality)[a] | Overall Z-score (Quality)[b] |
|---|---|---|---|---|---|---|---|---|
| **0** | dimer | 3ABI[9], 2AXQ[10], **4INA**[c], 1E5Q[11], 4RL6[c] | None | NO | NO | 25 | -1.564 (Satisfactory) | *N.d* |
| **1** | dimer | 1E5Q[11], **3ABI**[9], 2AXQ[10], 3IC5[c], 3WYE[c] | 4INA[c], 4RL6[c] | NADH | NO | 21 | -1.321 (Satisfactory) | *N.d* |
| **2** | monomer | 1E5Q[11], **3ABI**[9], 2AXQ[10], | 4INA[c], 4RL6[c] | NADH | NO | 15 | -1.161 (Satisfactory) | -1.11 (Satisfactory) |
| **2.1** | monomer | *N.a* | *N.a* | NADH | **1c** | *N.a* | *N.a* | -1.14 (Satisfactory) |
| **2.2** | monomer | *N.a* | *N.a* | NADH | **2c** | *N.a* | *N.a* | -1.18 (Satisfactory) |

[a].Z-score and quality of the model after hybrid model generation.
[b].Z-score and quality of the model after molecular dynamic refinement.
[c].Crystal structure not yet associated to a publication. Please visit the www.pdb.org website for more information.
N.d: Not determined
N.a: Not applicable

**Supplementary Table 13. Primers that have been used for preparing the variants**

| Primer name | Sequence |
|---|---|
| F173A_for | CTCTGGAATATAATCATGTTGCCAGCCTGGAAGGTCTGCTGG |
| F173A_rev | CCAGCAGACCTTCCAGGCTGGCAACATGATTATATTCCAGAG |
| F173G_for | CTGGAATATAATCATGTTGGTAGCCTGGAAGGTCTGCTGG |
| F173G_rev | CCAGCAGACCTTCCAGGCTACCAACATGATTATATTCCAG |
| V172A_173G_for | TCTGGAATATAATCATGCTGGTAGCCTGGAAGGTC |
| V172A_173G_rev | GACCTTCCAGGCTACCAGCATGATTATATTCCAGA |
| V172G_F173G_for | TCTGGAATATAATCATGGTGGTAGCCTGGAAGGTC |
| V172G_F173G_rev | GACCTTCCAGGCTACCACCATGATTATATTCCAGA |
| V172G_F173A_for | TCTGGAATATAATCATGGTGCCAGCCTGGAAGGTC |
| V172G_F173A_rev | GACCTTCCAGGCTGGCACCATGATTATATTCCAGA |
| V172A_F173A_for | TCTGGAATATAATCATGCTGCCAGCCTGGAAGGTC |
| V172A_F173A_rev | GACCTTCCAGGCTGGCAGCATGATTATATTCCAGA |
| R242M_for | CGTCTGGAATACAAAACCATTATGTATCGTGGTCATGCCGAAAAATG |
| R242M_rev | CATTTTTCGGCATGACCACGATACATAATGGTTTTGTATTCCAGACG |
| R242W_for | CTGGAATACAAAACCATTTGGTATCGTGGTCATGCCGAAAAATG |
| R242W_rev | CATTTTTCGGCATGACCACGATACCAAATGGTTTTGTATTCCAG |
| V130A_for | TTCCGGATCTGGGCGCTGCACCGGGTAT |
| V130A_rev | ATACCCGGTGCAGCGCCCAGATCCGGAA |
| V130G_for | TTCCGGATCTGGGCGGTGCACCGGGTAT |
| V130G_rev | ATACCCGGTGCACCGCCCAGATCCGGAA |
| F173V_for | CTGGAATATAATCATGTTGTCAGCCTGGAAGGTCTG |
| F173V_rev | CAGACCTTCCAGGCTGACAACATGATTATATTCCAG |

**Supplementary Table 14**. Results for the biocatalytic reactions performed by ωTAs for compounds **9a**-**13a**.

| Substrate | Enzyme | Recovered substrate (%) | Amine product[a] Conv (%) | Alcohol (%) | Reported Selectivity |
|---|---|---|---|---|---|
| 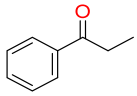 **9a** | As-ωTA | 94 | 6 | - | *(R)* |
| | At-ωTA | 97 | 3 | - | *(R)* |
| | Hn-ωTA | >99 | - | - | *(R)* |
| | Ac-ωTA | 98 | 2 | - | *(S)* |
| | Bm-ωTA | 3 | 97 | - | *(S)* |
| | Cv-ωTA | 70 | 30 | - | *(S)* |
| | Pd-ωTA | 47 | 53 | - | *(S)* |
| | Pf-ωTA | 47 | 53 | - | *(S)* |
| 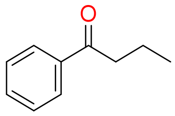 **10a** | As-ωTA | 100 | - | - | *(R)* |
| | At-ωTA | 100 | - | - | *(R)* |
| | Hn-ωTA | 100 | - | - | *(R)* |
| | As*R*mut11-ωTA | 85 | 15 | - | *(R)* |
| | Ac-ωTA | 100 | - | - | *(S)* |
| | Bm-ωTA | 87 | 13 | - | *(S)* |
| | Cv-ωTA | >99 | <1 | - | *(S)* |
| | Pd-ωTA | 96 | 4 | - | *(S)* |
| | Pf-ωTA | >99 | <1 | - | *(S)* |
| 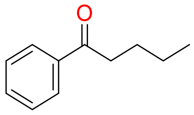 **11a** | Vf-ωTA | 6 | 14 | - | *(S)* |
| | As-ωTA | 100 | - | - | *(R)* |
| | At-ωTA | 100 | - | - | *(R)* |
| | Hn-ωTA | 100 | - | - | *(R)* |
| | As*R*mut11-ωTA | 97 | 3 | - | *(R)* |
| | Ac-ωTA | 100 | - | - | *(S)* |
| | Bm-ωTA | 92 | 8 | - | *(S)* |
| | Cv-ωTA | >99 | <1 | - | *(S)* |
| | Pd-ωTA | 99 | 1 | - | *(S)* |
| 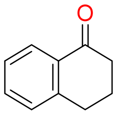 **12a** | As*R*mut11-ωTA | 52 | 48 | - | *(R)* |
| | Vf-ωTA | 32 | 68 | - | *(S)* |
| 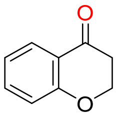 **13a** | As*R*mut11-ωTA | 26 | 74 | - | *(R)* |
| | Vf-ωTA | 69 | 25 | 6 | *(S)* |

[a] Conversions were determined using a 7890A GC system (Agilent Technologies), equipped with FID detector using $H_2$ as carrier gas with a DB-1701 column from Agilent (30 m, 250 µm, 0.25 µm)

**Supplementary Table 15**. Results for the biocatalytic reactions performed by ωTAs for compounds **16a-19a**.

| Substrate | Enzyme | Recovered substrate (%) | Amine product[a] Conv (%) | Alcohol (%) | Reported Selectivity |
|---|---|---|---|---|---|
| 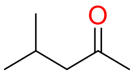 **16a** | As-ωTA | 100 | - | - | *(R)* |
| | At-ωTA | 100 | - | - | *(R)* |
| | Hn-ωTA | 73 | 27 | - | *(R)* |
| | Ac-ωTA | 89 | 11 | - | *(S)* |
| | Bm-ωTA | 44 | 56 | - | *(S)* |
| | Cv-ωTA | 11 | 89 | - | *(S)* |
| | Pd-ωTA | 89 | 11 | - | *(S)* |
| | Pf-ωTA | 66 | 34 | - | *(S)* |
| | Vf-ωTA | 77 | 23 | - | *(S)* |
| 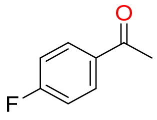 **17a** | As-ωTA | 13 | 85 | 2 | *(R)* |
| | At-ωTA | 83 | 11 | 6 | *(R)* |
| | Hn-ωTA | 90 | 1 | 8 | *(R)* |
| | Ac-ωTA | 48 | 47 | 5 | *(S)* |
| | Bm-ωTA | 16 | 80 | 4 | *(S)* |
| | Cv-ωTA | 7 | 91 | 2 | *(S)* |
| | Pd-ωTA | 4 | 93 | 3 | *(S)* |
| | Pf-ωTA | 43 | 50 | 7 | *(S)* |
| | Vf-ωTA | 2 | 97 | 1 | *(S)* |
| 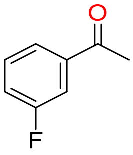 **18a** | As-ωTA | 3 | 96 | 1 | *(R)* |
| | At-ωTA | 27 | 70 | 3 | *(R)* |
| | Hn-ωTA | 76 | 7 | 17 | *(R)* |
| | Ac-ωTA | 21 | 75 | 4 | *(S)* |
| | Bm-ωTA | 13 | 81 | 6 | *(S)* |
| | Cv-ωTA | 2 | 97 | 1 | *(S)* |
| | Pd-ωTA | 1 | 98 | 1 | *(S)* |
| | Pf-ωTA | 14 | 82 | 4 | *(S)* |
| | Vf-ωTA | 1 | 98 | 1 | *(S)* |
| 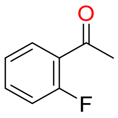 **19a** | As-ωTA | 1 | 98 | 1 | *(R)* |
| | At-ωTA | 2 | 96 | 2 | *(R)* |
| | Hn-ωTA | 61 | 19 | 20 | *(R)* |
| | Ac-ωTA | 11 | 85 | 4 | *(S)* |
| | Bm-ωTA | 3 | 93 | 4 | *(S)* |
| | Cv-ωTA | <1 | 99 | <1 | *(S)* |
| | Pd-ωTA | 1 | 99 | - | *(S)* |
| | Pf-ωTA | 14 | 76 | 10 | *(S)* |
| | Vf-ωTA | <1 | 99 | - | *(S)* |

[a] Conversions were determined using a 7890A GC system (Agilent Technologies), equipped with FID detector using $H_2$ as carrier gas with a DB-1701 column from Agilent (30 m, 250 µm, 0.25 µm)

**Supplementary Table 16**. Results for the biocatalytic reactions performed by ωTAs for compounds **20a** and **22a**.

| Substrate | Enzyme | Recovered substrate (%) | Amine product[a] Conv (%) | Alcohol (%) | Reported Selectivity |
|---|---|---|---|---|---|
| 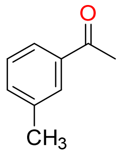 **20a** | As-ωTA | 13 | 85 | 2 | *(R)* |
| | At-ωTA | 27 | 71 | 2 | *(R)* |
| | Hn-ωTA | 86 | 9 | 5 | *(R)* |
| | Ac-ωTA | 87 | 8 | 5 | *(S)* |
| | Bm-ωTA | 83 | 8 | 9 | *(S)* |
| | Cv-ωTA | 4 | 93 | 3 | *(S)* |
| | Pd-ωTA | 66 | 25 | 9 | *(S)* |
| | Pf-ωTA | 7 | 90 | 3 | *(S)* |
| | Vf-ωTA | 13 | 85 | 2 | *(S)* |
| 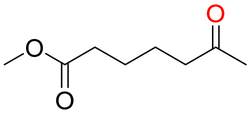 **22a** | As-ωTA | 10 | 90 | - | *(R)* |
| | At-ωTA | 27 | 73 | - | *(R)* |
| | Hn-ωTA | 69 | 31 | - | *(R)* |
| | Ac-ωTA | 63 | 37 | - | *(S)* |
| | Bm-ωTA | 32 | 68 | - | *(S)* |
| | Cv-ωTA | 12 | 88 | - | *(S)* |
| | Pf-ωTA | 86 | 14 | - | *(S)* |
| | Vf-ωTA | 32 | 68 | - | *(S)* |

[a] Conversions were determined using a 7890A GC system (Agilent Technologies), equipped with FID detector using $H_2$ as carrier gas with a DB-1701 column from Agilent (30 m, 250 µm, 0.25 µm)

**Supplementary Table 17**. Melting temperature $T_m$ of LE-AmDH-v1 at different pH values. Error bars represent the standard deviation from n=3 independent experiments. Source data are provided as a Source Data file. ± is SD (n=3).

| | | Tm (°C) | |
|---|---|---|---|
| **buffer** | | + **8a** | + $NAD^+$ |
| 2 M $HCOONH_3/NH_4$ pH 7.5 | 66 ± <1 | N.m. | N.m. |
| 2 M $HCOONH_3/NH_4$ pH 8.0 | 67 ± <1 | N.m. | N.m. |
| 2 M $HCOONH_3/NH_4$ pH 8.5 | 67 ± <1 | N.m. | N.m. |
| 2 M $HCOONH_3/NH_4$ pH 9.0 | 67± <1 | 67 ± <1 | 69 ± <1 |
| 2 M $HCOONH_3/NH_4$ pH 9.5 | 66 ± <1 | N.m. | N.m. |

**Supplementary Table 18**. Determination of the saturation concentration for NADH with LE-AmDH-v1 or Ch1-AmDH at 60 °C.

| enzyme | $K_M$ (µM) | conc. NADH employed for steady state kinetics (µM) |
|---|---|---|
| Le-AmDH-v1 | 42.3 ± 1.7 | 250 |
| Ch1-AmDH | 19.9 ± 1.6 | 250 |

**Supplementary Table 19**. Kinetic assay for the determination of the Steady-state kinetics for LE-AmDH-v1 and Ch1-AmDH.

| enzyme | substrate | substrate (mM) | Temp (°C) | enzyme (µM) |
|---|---|---|---|---|
| LE-AmDH-v1 | **8a** | 0-30[a] | 60 | 5.2 |
| | **8a** | 0-30[a] | 50 | 5.2 |
| | **8a** | 0-30[a] | 40 | 8.0 |
| Ch1-AmDH | **8a** | 0-30[a] | 60 | 2.0 |
| | **8a** | 0-30[a] | 50 | 2.0 |
| | **8a** | 0-30[a] | 40 | 3.2 and 4.2 |
| LE-AmDH-v1 | **21a** | 0-15[b] | 60 | 1.2 |

[a] 500 mM main stock in DMSO which was further diluted to 350 mM in buffer
[b] Directly added as 500 mM stock in DMSO; solubility problems at higher substrate concentrations

**Supplementary Table 20**. Inhibition studies for LE-AmDH-v1 and Ch1-AmDH at 15 mM **8a** with (*R*)-**8b** as inhibitor.

| enzyme | $IC_{50}$ (mM) |
|---|---|
| LE-AmDH-v1 | 20 |
| Ch1-AmDH | 1 |

**Supplementary Table 21**. Kinetic parameter obtained in the inhibition study for **8a**. (fit according to Michaelis Menten equation).

| enzyme | (*R*)-**8b** [mM] | $k_{app}$ (min$^{-1}$) | $K_{M,app}$ (mM) | $k_{app}/K_{M,app}$ (M min$^{-1}$) |
|---|---|---|---|---|
| LE-AmDH-v1 | 0 | 7.1 ± 0.2 | 5.5 ± 0.5 | 1282 |
| | 5 | 5.8 ± 0.3 | 7.8 ± 1.0 | 749 |
| | 10 | 5.1 ± 0.4 | 10.3 ± 1.6 | 498 |
| Ch1-AmDH | 0 | 17.7 ± 0.4 | 5.6 ± 0.4 | 3180 |
| | 0.2 | 17.0 ± 0.6 | 10.8 ± 0.8 | 1580 |
| | 0.4 | 14.5 ± 0.6 | 15.4 ± 1.4 | 940 |

**Supplementary Table 22**. $K_i$ Parameter determined from the linear fits of the double reciprocal plots according to Lineweaver-Burk.

| enzyme | (*R*)-**8b** [mM] | slope | intercept |
|---|---|---|---|
| LE-AmDH-v1 | 0 | 0.86765 | 0.13121 |
| | 5 | 1.82492 | 0.11888 |
| | 10 | 2.79835 | 0.11547 |
| Ch1-AmDH | 0 | 0.28621 | 0.05956 |
| | 0.2 | 0.71526 | 0.05158 |
| | 0.4 | 1.2071 | 0.05905 |

**Supplementary Table 23**. Results obtained from Equation 1 considering different initial concentrations of substrate.

| enzyme | $K_M^{eff}$ $[S_0]$ = 10 mM | $K_M^{eff}$ $[S_0]$ = 50 mM | $K_M^{eff}$ $[S_0]$ = 100 mM | $k_{app}/K_M^{eff}$ (M $min^{-1}$) $[S_0]$ = 10 mM | $k_{app}/K_M^{eff}$ (M $min^{-1}$) $[S_0]$ = 50 mM | $k_{app}/K_M^{eff}$ (M $min^{-1}$) $[S_0]$ = 100 mM |
|---|---|---|---|---|---|---|
| Le-AmDH-v1 | 69 | 246 | 467 | 102 | 29 | 15 |
| Ch1-AmDH | 1707 | 8432 | 16838 | 10 | 2 | 1 |

**Supplementary Table 24**. Experimentally determined *ee* values

| | | GC Area (*R*) | GC Area (S) | % (R) | % (S) | ***ee* [%]** |
|---|---|---|---|---|---|---|
| (*R*)-**8b** | reference compound | 19584 | 97 | 99.51 | 0.49 | 99.0 |
| (*S*)-**8b** | reference compound | 82 | 19421 | 0.42 | 99.58 | 99.2 |
| (*R*)-**8b** | preparative scale | 16473 | n.d.[a] | >99.98 | <0.012 | **>99.9** |
| (*R*)-**9b** | reference compound | 6909 | 91 | 98.70 | 1.3 | 97.4 |
| (*S*)-**9b** | reference compound | 31 | 12038 | 0.26 | 99.74 | 99.5 |
| (*R*)-**9b** | preparative scale | 8492 | n.d.[a] | >99.97 | <0.023 | **>99.9** |

[a] For calculation of the *ee*, a GC peak area of 2 was used as threshold value, as this is clearly detectable with our equipment.

## Supplementary Methods

### Homology modelling generation

The generation of homology models was carried out using the YASARA[1] homology model building protocol[2], which involves multi-template structural model generation. Since the linear amino acid sequence of the target protein was the only given input, the possible templates were identified by running 3 PSI-BLAST[3] iterations to extract a position specific scoring matrix (PSSM) from UniRef90[4], and then searching the PDB for a match with an E-value below the homology modelling cut-off 0.005. A maximum of 5 templates was allowed. To aid alignment correction and loop modelling, a secondary structure prediction for the target sequence had to be obtained. This was achieved by running PSI-BLAST to create a target sequence profile and feeding it to the PSI-Pred[5] secondary structure prediction algorithm. For each of the found templates, models were built. Either a single model per template was generated, when the alignment was certain, or a number of alternative models were generated, when the alignment was ambiguous. A maximum of 100 conformations per loop were explored. A maximum of 10 residues were added to the termini. Finally, YASARA tried to combine the best parts of the generated models to obtain a hybrid model, with the intension of increasing the accuracy beyond each of the contributors. The quality of the models was evaluated by use of Z-score[6,7]. A Z-score describes how many standard deviations the model quality is away from the average high-resolution X-ray structure. The overall Z-scores for all models have been calculated as the weighted averages of the individual Z-scores using the formula:

**Supplementary equation 1** Overall = 0.145*Dihedrals + 0.390*Packing1D + 0.465*Packing3D

The overall score thus captures the correctness of backbone- (Ramachandran plot) and side-chain dihedrals, as well as packing interactions. In order to increase their quality, the obtained models were submitted to 500 ps molecular dynamic refinement simulation using the protocol describe by E. Krieger *et al.*[8] A structural snapshot was saved every 25 ps for further analysis of quality parameters (potential energy, Dihedrals, Packing1D and Packing3D).

Amino acid sequence of the wild-type L-lysine epsilon-dehydrogenase from *Geobacillus stearothermophilus* (LysEDH):

```
MKVLVLGAGLMGKEAARDLVQSQDVEAVTLADVDLAKAEQTVRQLHSKKLAAVRVDAGDPQQLAAAMKGHD
VVVNALFYQFNETVAKTAIETGVHSVDLGGHIGHITDRVLELHERAQAAGVTIIPDLGVAPGMINILSGYG
ASQLDEVESILLYVGGIPVRPEPPLEYNHVFSLEGLLDHYTDPALIIRNGQKQEVPSLSEVEPIYFDRFGP
LEAFHTSGGTSTLSRSFPNLKRLEYKTIRYRGHAEKCKLLVDLTLTRHDVEVEINGCRVKPRDVLLSVLKP
LLDLKGKDDVVLLRVIVGGRKDGKETVLEYETVTFNDRENKVTAMARTTAYTISAVAQLIGRGVITKRGVY
PPEQIVPGDVYMDEMKKRGVLISEKRTVHSLE
```

The first homology model generation delivered model 0, which does not contain cofactor or ligand in its active site. Therefore, a second run was carried out excluding templates 4INA, 4RL6 for not containing cofactor or confirmation of the desired activity. This run yielded model 1 (dimeric) containing only the cofactor NADH but not ligand. In order to eliminate possible crystal contact artefacts, a third run was carried out also excluding templates 4INA, 4RL6 and allowing only the monomeric model to be generated. This run produced model 2 that contains the desired cofactor. In order to include the substrate, the intermediate model created with template 1E5Q, which contains not only the cofactor but also a ligand (saccharopine), was combined with model 2 and saccharopine was *in silico* modified into compound **1c** (model 2.1) and subsequently into **2c** (model 2.2) (**Supplementary Figure 21**). These three last models (2, 2.1 and 2.2 from **Supplementary Table 12**) were submitted to molecular dynamic refinement.

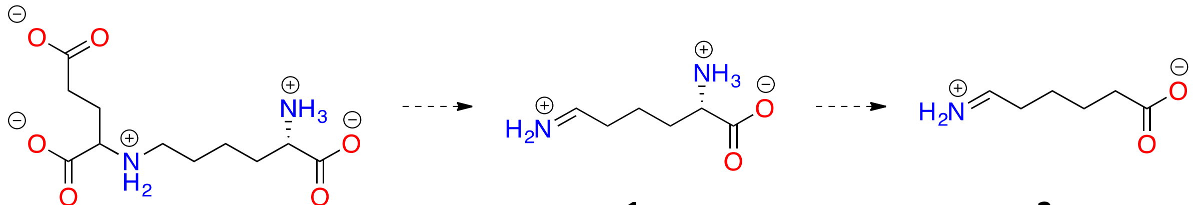


**Supplementary Figure 21**. **Schematic representation of the *in-silico* transformation of saccharopine.** Saccharopine was transformed first to compound 1c and then into 2c in the active site of the final homology model

## Molecular dynamics (MD) simulations

MD simulations were executed using the Yasara software[12], with the AMBER03 force field[13]. In order to generate equilibrated starting structures for the MD simulations, each system (containing the desired mutations, co-factor and substrate) was placed in a rectangular box of simple point charge (SPC) water, to which approximately 100 mM NaCl (including neutralizing counter-ions) was added to simulate physiological salinity[14]. The protonation state of all atoms was automatically adjusted, with the exception of those atoms involved in the hydride transfer between co-factor and the substrate which were usually inspected and manually altered accordingly. The protonation state of the histidine residues and the orientation of asparagine and glutamine residues was automatically determined by its environment (hydrogen-bond network) using the graph-theory algorithm included in Yasara[15-17]. The walls of the simulation box were located at least 5.0 Å from the surface of the protein, following energy minimization (steepest descents) where α-carbon atoms, co-factor and substrate were kept frozen. Each of the systems was equilibrated in two-step protocol, with position restraints applied to the protein heavy atoms throughout, co-factor and substrate. The first phase involved simulating for 100 ps under a constant volume (NVT) ensemble. The complete system was coupled to temperature coupling baths, and temperature was maintained at 298 K using the V-rescale coupling method[18]. Following NVT equilibration (phase 2 of the equilibration), 500 ps of constant pressure (NPT) equilibrations were performed, also using weak coupling to maintain pressure isotropically at 1.0 bar. All productions MD simulations were carried out under NPT conditions with 2.5 fs step size (YASARA fast protocol)[19]. The LINCS algorithm[20] was used to constrain the lengths of hydrogen containing bonds; the waters were restrained using the SETTLE algorithm[21]. Van der Waals forces were treated using a 12 Å cut-off. Long-range electrostatic forces were treated using the particle mesh Ewald method (PME)[22]. Both binding conformations, pro-*R* and pro-*S*, were submitted to MD simulations. A minimum of six independent MD simulations (with random initial velocities) were executed per system. Each MD simulation was allowed to run for 500 ps, and a snapshot was taken every 6.25 ps, thus resulting in 81 frames (counting the starting structure) per simulation (**Supplementary Figures 22-23**). These frames were submitted for analysis and several dynamic properties were followed

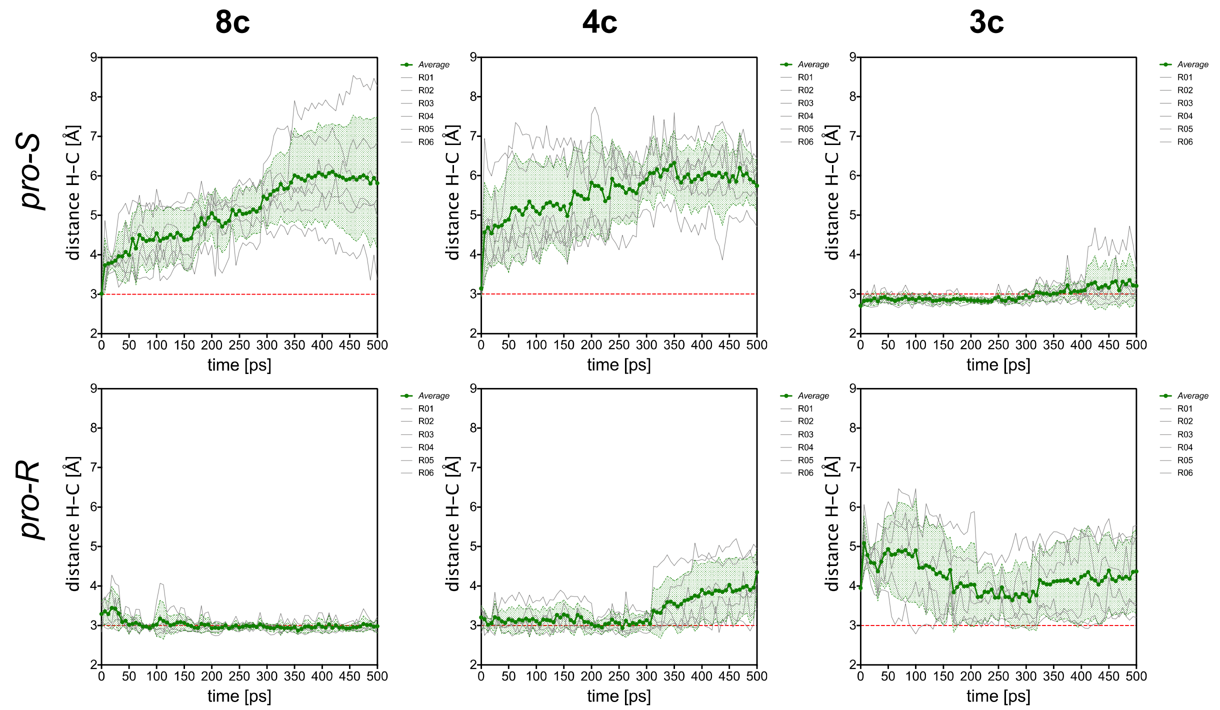


**Supplementary Figure 22**. **Distance hydride/pro-chiral carbon vs. time for substrates 8c, 4c, and 3c**. The distance H-C is the main parameter determining the putative reactivity of this enzyme towards a given substrate. All independent simulations are shown in grey, while the average of all these independent simulations is shown in green connected dots. The standard deviation of all n=6 independent simulations is depicted as a green area around the average.

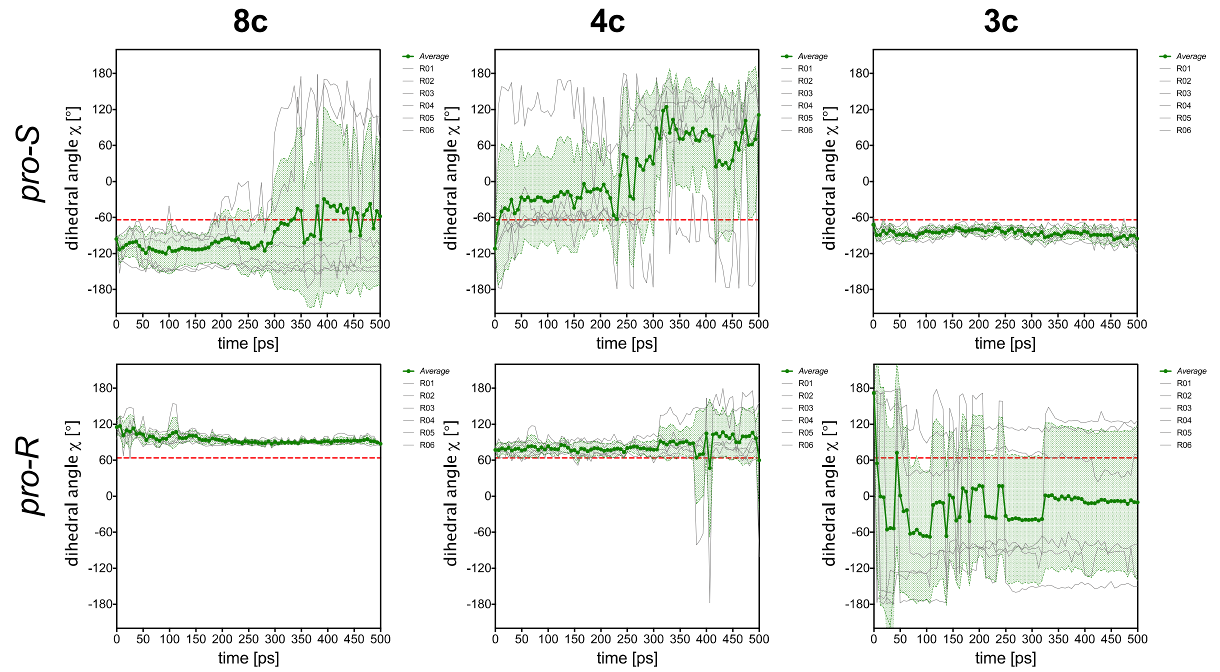


**Supplementary Figure 23. Dihedral angle χ vs. time for substrates 8c, 4c, and 3c**. The dihedral angle χ is the main parameter determining the putative stereo-selectivity of this enzyme towards a given substrate. All independent simulations are shown in grey, while the average of all these independent simulations is shown in green connected dots. The standard deviation of all n=6 independent simulations is depicted as a green area around the average. This dihedral angle for the *R*- and *S*-configured products of the reaction, showed to have an average value of 63.88 ± 0.21°, and -63.97 ± 0.29°, respectively. These reference values are here depicted with a red dashed-line.

## Site directed mutagenesis of LysEDH variants

The LysEDH variants were obtained by site directed mutagenesis using the QuickChange II Site-Directed Mutagenesis Kit (Agilent Technologies) according to the protocol of the supplier and by using following template DNA (subcloned into pET28b). The primers used are shown in **Supplementary Table 13.**

```
ATGAAAGTTCTGGTTCTGGGTGCAGGTCTGATGGGTAAAGAAGCAGCACGCGATCTGGTTCAGAGCCAGGA
TGTTGAAGCAGTTACCCTGGCAGATGTTGATCTGGCAAAAGCAGAACAGACCGTTCGTCAGCTGCATAGCA
AAAAACTGGCAGCAGTTCGTGTTGATGCCGGTGATCCGCAGCAGCTGGCAGCCGCAATGAAAGGTCATGAT
GTTGTTGTTAATGCCCTGTTCTATCAGTTTAATGAAACCGTTGCAAAAACCGCCATTGAAACCGGTGTTCA
TAGCGTGGATCTGGGTGGTCATATTGGCCATATTACCGATCGTGTTCTGGAACTGCATGAACGTGCACAGG
CAGCCGGTGTTACCATTATTCCGGATCTGGGCGTTGCACCGGGTATGATTAACATTCTGAGCGGTTATGGT
GCAAGCCAGCTGGATGAAGTTGAAAGCATTCTGCTGTATGTTGGTGGTATTCCGGTTCGTCCGGAACCGCC
TCTGGAATATAATCATGTTTTTAGCCTGGAAGGTCTGCTGGATCATTATACCGATCCGGCACTGATTATTC
GTAATGGTCAGAAACAAGAAGTTCCGAGCCTGAGCGAAGTTGAACCGATCTATTTTGATCGTTTTGGTCCG
CTGGAAGCATTTCATACCAGCGGTGGCACCAGCACCCTGAGCCGTAGCTTTCCGAATCTGAAACGTCTGGA
ATACAAAACCATTCGTTATCGTGGTCATGCCGAAAAATGTAAACTGCTGGTTGATCTGACCCTGACACGCC
ATGATGTGGAAGTGGAAATTAATGGTTGTCGTGTTAAACCGCGTGATGTTCTGCTGAGCGTTCTGAAACCG
CTGCTGGACCTGAAAGGTAAAGATGATGTGGTTCTGCTGCGTGTTATTGTGGGTGGTCGTAAAGATGGTAA
AGAAACAGTGCTGGAATATGAAACGGTGACCTTTAATGATCGCGAAAATAAAGTTACCGCAATGGCACGTA
CCACCGCATATACCATTAGCGCAGTTGCACAGCTGATTGGTCGTGGTGTTATTACCAAACGTGGTGTTTAT
CCGCCTGAGCAGATTGTTCCGGGTGATGTTTATATGGACGAAATGAAAAAACGCGGTGTGCTGATTAGCGA
AAAACGTACCGTTCATAGC
```

After verifying positive clones by sequencing, *E. coli* BL21 DE3 cells were transformed with the DNA and used as host for expression of the proteins.

## Protein expression purification and activity assays

Formate dehydrogenase from *Candida boidinii* (Cb-FDH) and Ch1-AmDH were expressed and purified as described previously.[23,24]

For heterologous expression of wild-type LysEDH and its variants, *E. coli* BL21(DE3) was used as host organism. 800 mL LB medium, supplemented with kanamycin (50 µg $mL^{-1}$) were inoculated with 15 mL of pre-culture and cells were allowed to grow at 37 °C until an $OD_{600}$ of 0.7-1 was reached. Expression of the enzyme was induced by the addition of 0.5 mM IPTG and performed overnight at 25 °C. The cells were harvested by centrifugation (4.500 g, 20 min, 4 °C), resuspended in lysis buffer ($KH_2PO_4$, 300 mM NaCl, 10 mM imidazole, pH 8.0) and lysed by sonication. The lysate was centrifuged (18.000 rpm, 50 min, 4 °C) and filtered through a 0.45 µm filter. Protein purification was performed by Ni-NTA affinity chromatography using pre-packed Ni-NTA His Trap FF columns (GE Healthcare) according to the manufacturer´s instruction. After loading of the filtered lysate, the column was washed with sufficient amounts of washing buffer (50 mM $KH_2PO_4$, 300 mM NaCl, 25 mM imidazole, pH 8.0), and bound protein was recovered with elution buffer (50 mM $KH_2PO_4$, 300 mM NaCl, 200 mM imidazole, pH 8.0). The process of purification was analysed by SDS-PAGE and fractions containing pure protein were pooled and dialyzed overnight against KPibuffer (50 mM, pH 8.0). Protein solutions were concentrated and the concentration was determined spectrophotometrically using the extinction coefficient at 280 nm. Typically, a protein yield of 80 mg pure protein per liter of cell culture was obtained. The purity of the enzymes was verified by SDS-PAGE (**Supplementary Figure 24**).

| | LysDH Variant | Mutations |
|---|---|---|
| **1** | LysEDH | *none* |
| **2** | LE-AmDH-v1 | *F173A* |
| **3** | LE-AmDH-v2 | *F173G* |
| **4** | LE-AmDH-v3 | *V172A, F173G* |
| **5** | LE-AmDH-v4 | *V172G, F173G* |
| **6** | LE-AmDH-v5 | *V172G, F173A* |
| **7** | LE-AmDH-v6 | *V172A, F173A* |
| **8** | LE-AmDH-v7 | *R242M, F173A* |
| **9** | LE-AmDH-v8 | *R242W, F173A* |
| **10** | LE-AmDH-v9 | *V130A, F173V* |
| **11** | LE-AmDH-v10 | *V130G, F173V* |
| **12** | LE-AmDH-v11 | *V130A, F173A* |
| **13** | LE-AmDH-v12 | *V130G, F173A* |
| **14** | LE-AmDH-v13 | *R242M, F173V* |
| **15** | LE-AmDH-v14 | *R242M* |

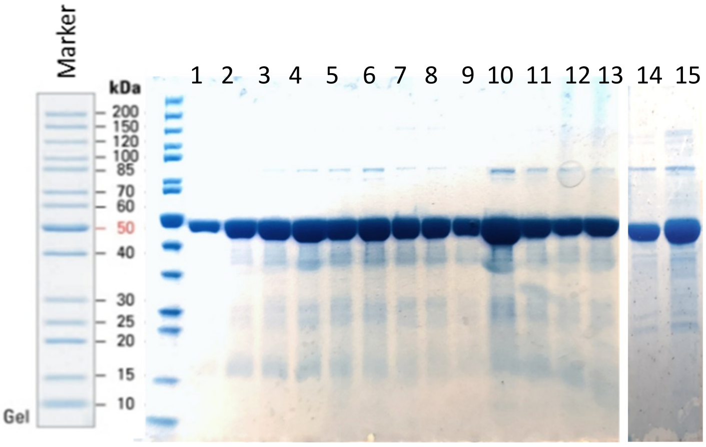


**Supplementary Figure 24**. **Purity of the enzymes used in this study**. All variants have similar molecular weights of approximately 45.000 Da. The mutations introduced to each variant are also shown on the left. SDS page: Source data are provided as a Source Data file.

The catalytic activity for the amination of benzaldehyde catalysed by LE-AmDH-v1 was in general ca. 730 mU mg$^{-1}$ during our study depending on the batch [measured for benzaldehyde (10 mM) in the ammonium buffer (2 M, pH 9.0) at 60 °C]. This assay was used for a practical and rapid comparison among the enzyme batches produced in this study.

The catalytic activity for the amination of acetophenone catalysed by LE-AmDH-v1 was in general ca. 200 mU mg$^{-1}$ [measured for acetophenone (30 mM) in the ammonium buffer (2 M, pH 9.0) at 60 °C].

The specific activity of the wild-type (ε-deaminating) L-lysine dehydrogenase towards L-lysine is known as it was determined by Heydari et al. in their original publication.[25] The reported value is 7.81 U mg$^{-1}$ [measured for L-lysine (10 mM) in glycine-KOH buffer (100 mM, pH 10), at 50 °C].

## Synthesis of 6-oxo-hexanoic acid (2a)

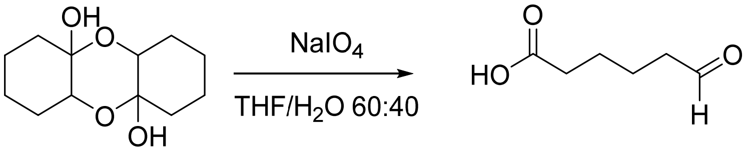


**Supplementary Figure 25**. **Synthesis of 2a.** The product was synthetized starting from 2-hydroxycyclohexanone dimer using 2.5 eq. of $NaIO_4$ and a mixtures of 60:40 tetrahydrofuran: water.

In a 250 mL one-neck round bottom flask, 2-hydroxycyclohexanone dimer (4.38 mmol, 1.00 g) and sodium periodate ($NaIO_4$, 10.95 mmol, 2.3 g, 2.5 eq.) were dissolved in tetrahydrofuran (THF, 60 mL) and $H_2O$ (40 mL, **Supplementary Figure 25)**. The mixture was stirred under magnetic agitation for 48 h at room temperature. By TLC analysis a single product was detected (hexane/ethyl acetate 60:40; $Rf_{product}$= 0.15). The reaction mixture was extracted with EtOAc (ethyl acetate, 3 x 50 mL), the combined organic layers were washed with a saturated solution of NaCl (3 x 50 mL), dried over $MgSO_4$ and concentrated under reduced pressure. An off-white solid was obtained in 87% yield (496 mg, 3.8 mmol) without any further purification. The product was analysed by $^1$H-NMR (**Supplementary Figure 26**, 400 MHz in $CDCl_3$) and compared with literature data[26].

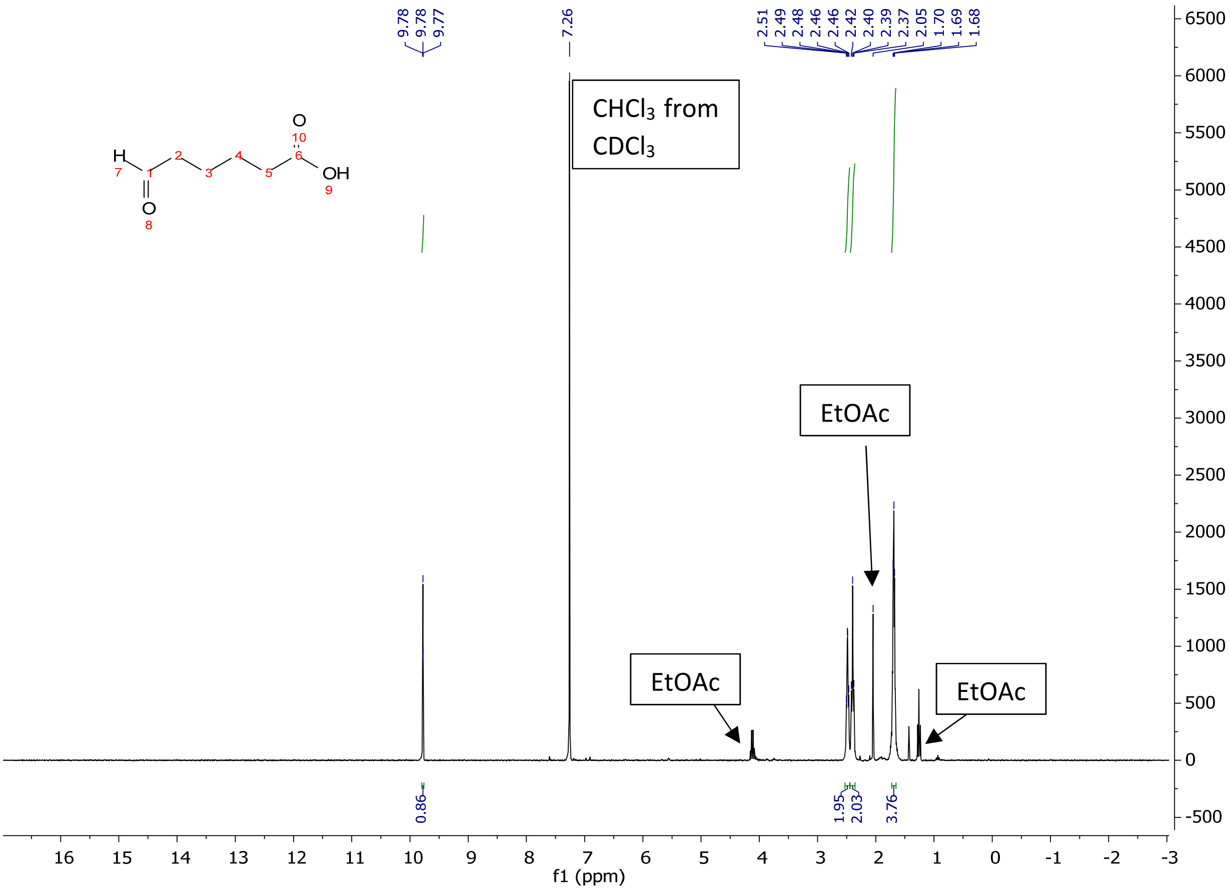


**Supplementary Figure 26. $^1$H-NMR spectrum of 2a**. The spectrum was recorded in $CDCl_3$ after isolation, without any further purification required, on a Bruker Avance 400 MHz NMR spectrometer.

### Synthesis of methyl-6-oxo-heptanoate (22a)

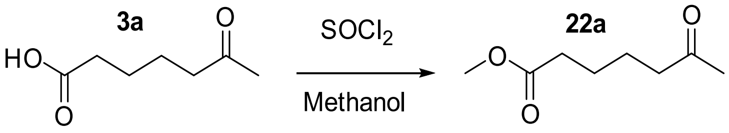


**Supplementary Figure 27. Synthesis of 22a.** Compound 22a was obtained in 83% yield after a simple methylation using thionyl chloride (2 eq.) as catalyst and methanol.

**3a** (100 mg, 0.7 mmol) was dissolved in methanol (3 mL) and the mixture was stirred in a round bottom flask on ice. Thionyl-chloride ($SOCl_2$, 2 eq., 167 mg, 1.406 mmol) was added dropwise and the mixture was heated up to 60 °C for 3 h ((**Supplementary Figure 27**). After that, GC-MS analysis (**Supplementary Figure 28**) confirmed quantitative conversion. The solvent was removed under reduced pressure affording the product as an orange/red oil (92 mg, 83% yield, MW = 158.19 g/mol).

A

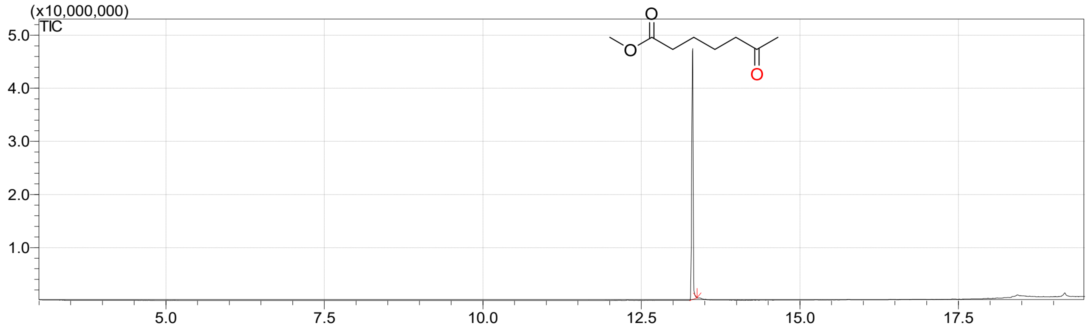


Mass spectrum of the peak at 13.3 min (**22a**)

B

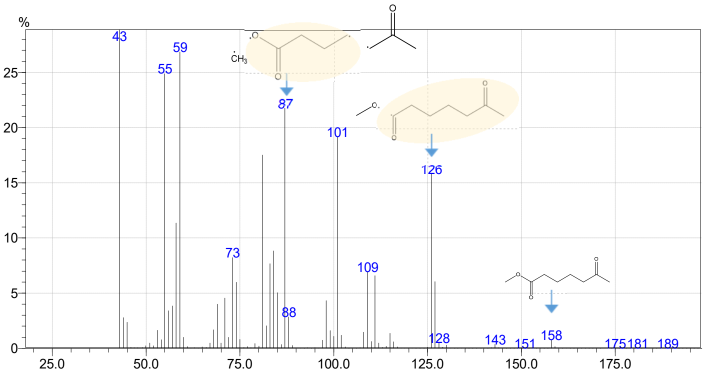


**Supplementary Figure 28. GC-MS analysis of 6-oxo-heptanoate.** (A) GC-MS chromatogram and (B) mass spectrum for the product **22a** obtained after esterification.

**Synthesis of enantiopure amines as reference compounds**

Synthesis of non-commercially available reference compounds was performed employing ω-transaminases (ωTAs)[27-29]. Three (*R*) selective ωTAs (*Hyphomonas neptunium* Hn-ωTA, *Aspergillus terreus* At-ωTA and *Arthrobacter sp.* As-ωTA), as well as six (*S*) selective ωTAs (*Bacillus megaterium* Bm-ωTA, *Chromobacterium violaceum* Cv-ωTA, *Paracoccus denitrificans* Pd-ωTA, *Pseudomonas fluorescens* Pf-ωTA, *Vibrio fluvialis* Vf-ωTA and *Arthrobacter citreus* Ac-ωTA) were used according to **Supplementary Figure 29.**

The (*R*) selective ωTA: As*R*mut11[28] was employed for the reductive amination of **10a-13a** according to **Supplementary Figure 30**.

Results are summarized in the **Supplementary Tables 14-16.**

For all ωTAs except As*R*mut11-ωTA, we incubated substrate (50 mM or 10 mM), alanine (250 mM, D-alanine for (*R*) selective ωTAs and L-alanine for (*S*) selective ωTAs), KPi buffer (pH 7.0, 100 mM), PLP (1 mM), freeze-dried *E. coli* cells containing overexpressed ωTA (20 mg), LDH (LDH-101, 60 U mg$^{-1}$), GDH (GDH-901, 50 U mg$^{-1}$), NAD$^+$ (1 mM), glucose (150 mM); reaction volume: 1 mL; reaction time: 24 h; temperature: 30 °C; agitation on an orbital shaker at 170 rpm.

For As*R*mut11-ωTA, we incubated $H_2O$, 2-propylamine (1 M), PLP (0.5 mM), adjusted to pH 11.0 with aqueous HCl, freeze-dried *E. coli* cells containing overexpressed ωTA (20 mg), substrate (50 mM or 10 mM). Reaction volume: 1 mL; reaction time: 24 h; temperature: 45 °C; agitation on an orbital shaker 170 rpm.

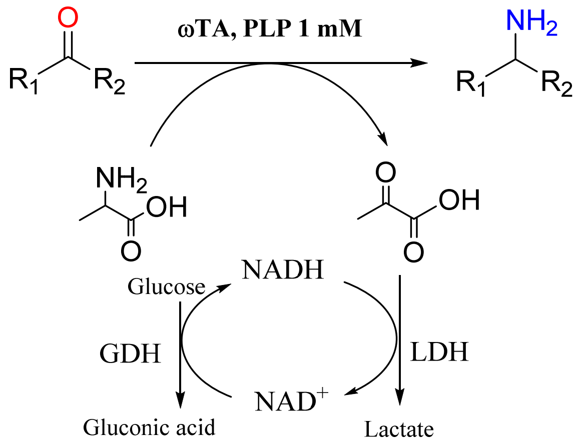


**Supplementary Figure 29**. **Schematic representation of the biocatalytic reactions performed by ωTAs.** This methodology was applied with all R- and S-selective ωTAs used in this study with the exception of the R-selective AsRmut11- ωTA.

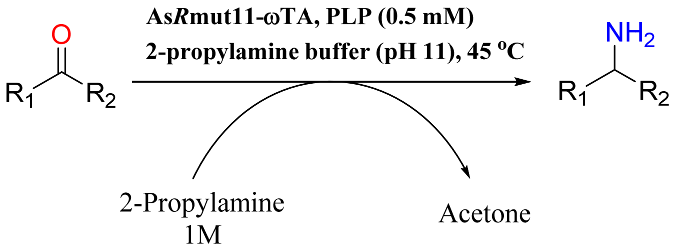


**Supplementary Figure 30. Schematic representation of the biocatalytic reactions performed by As*R*mut11 ωTA**. This methodology was applied using the R-selective AsRmut11-ωTA.

**Chemoenzymatic synthesis of methyl-6-aminoheptanonate (22b)**

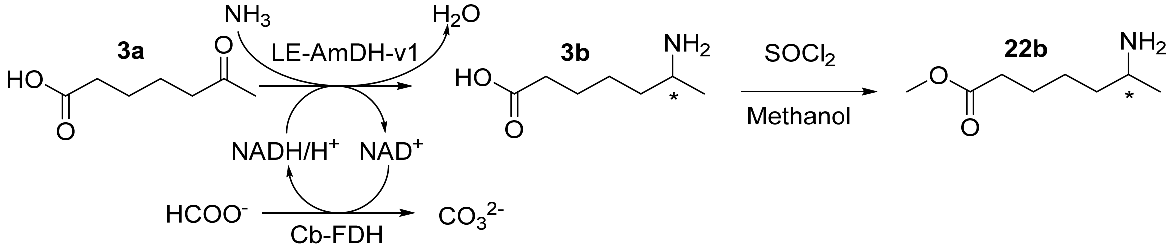


**Supplementary Figure 31**. **Schematic representation of the chemoenzymatic synthesis of 22b.** First step was performed using the LE-AmDH-v1 and after purification of compound 3b using ion exchange chromatography, a simple methylation reaction was performed to afford compound 22b

For Step 1 (**Supplementary Figure 31**), that is the conversion of **3a** to **3b**, the biocatalytic reaction (21 mL final volume) consisted of $HCOONH_4/NH_3$ (1 M, pH 9.0), LE-AmDH-v1 (90 µM), $NAD^+$ (1 mM), Cb-FDH (19 µM) and **3a** (50 mM, 150 mg, 1.040 mmol). The reaction was run at 30 °C for 48 h in an orbital shaker (170 rpm) before quenching by the addition of concentrated HCl (to pH 1). Aliquots of 2 mL were centrifuged in Eppendorf tubes (14.800 rpm, 20 min). The pellets were re-dissolved in water, centrifuged (14.800 rpm, 20 min) and all supernatants combined and freeze-dried overnight. The dry product was dissolved in HCl (0.1 M, 9 mL) and purified by cation exchange chromatography using a Dowex ® 50WX8 hydrogen form 100-200 mesh resin (Sigma Aldrich).

The resin (3 mL) was equilibrated by washing with 4 column columns (CV) of HCl (1 M, 12 mL), followed by 10 CV of HCl (0.1 M, 30 mL). The sample was loaded on the column and washed with $H_2O$ (5 CV, 15 mL). Elution of **3b** was performed with $NH_4OH$ (10%, 20 mL) in 5 mL aliquots. Fractions were freeze dried overnight and dissolved in water. Fractions containing the product were verified by TLC (ninhydrin staining, violet colour), combined and freeze dried. Pure **3b** was obtained as white solid in 73% yield (110 mg, 0.757 mmol).

For Step 2, that is the conversion of **3b** to **22b**, 75 mg (0.516 mmol) of **3b** obtained from step 1 were dissolved in methanol (3 mL) and HCl (2 M, ~150 µL) in a round bottom flask and the mixture was stirred on ice. Thionylchloride ($SOCl_2$, 2 eq., 1.03 mmol, 74.8 µL) was added dropwise and the reaction stirred at room temperature for 8 h. MeOH was removed under reduced pressure affording the crude product as an orange/red oil. $K_2CO_3$ (saturated solution, 3.5 mL) was added, thoroughly mixed, and the product was extracted with dichloromethane (DCM) (3 x 3 mL) and dried over $MgSO_4$. The solvent was removed under reduced pressure and **22b** was obtained as an orange oil in 73% yield (55 mg, 0.345 mmol).

The structures of **3b** before and after purification as well as of **22b** were confirmed by $^1$H-NMR (**Supplementary Figures 32-34**).

Finally, the enantiomeric excess of **22b** was determined to be >99% (*S*) by comparing its retention time with the retention times of the products obtained by enzymatic transformation of **22a** using the As-ωTA and Cv-ωTA after derivatization (as described in the main paper)

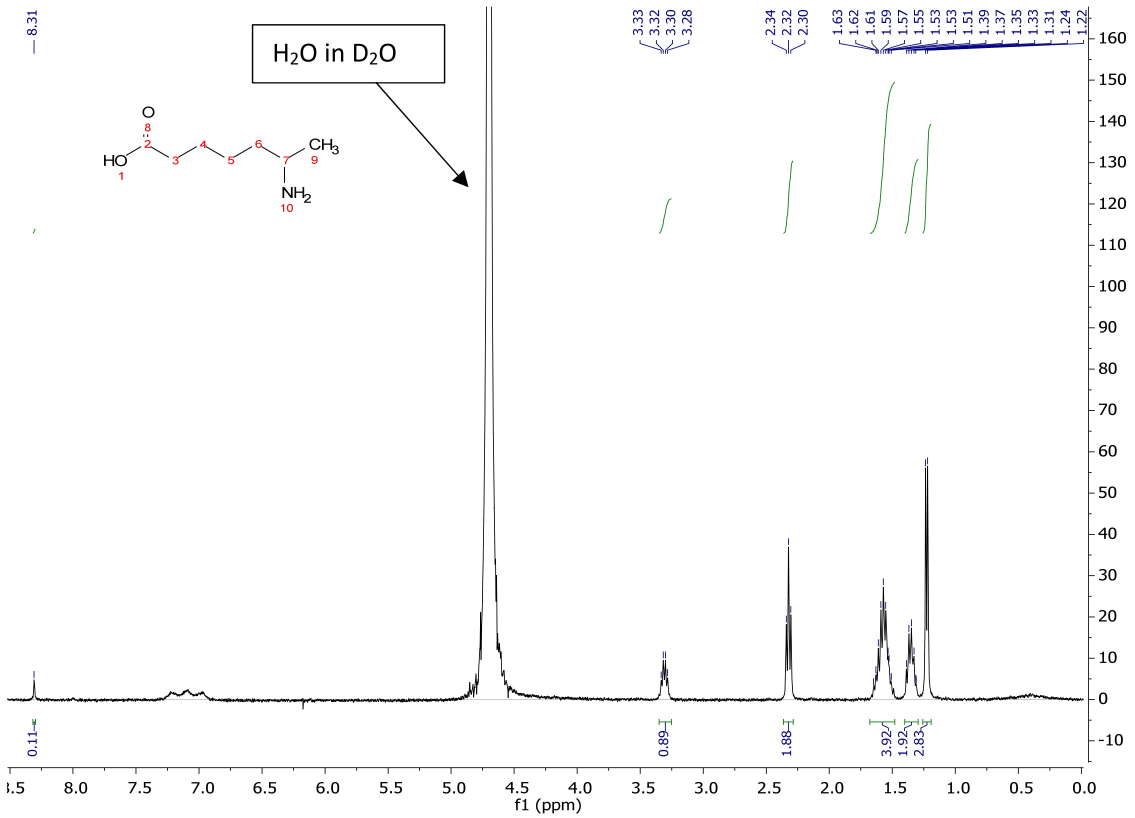


**Supplementary Figure 32**. **$^{1}$H-NMR spectrum of 3b.** The spectrum was recorded in $D_2O$ before purification with ion exchange chromatography on a Bruker Avance 400 MHz NMR spectrometer.

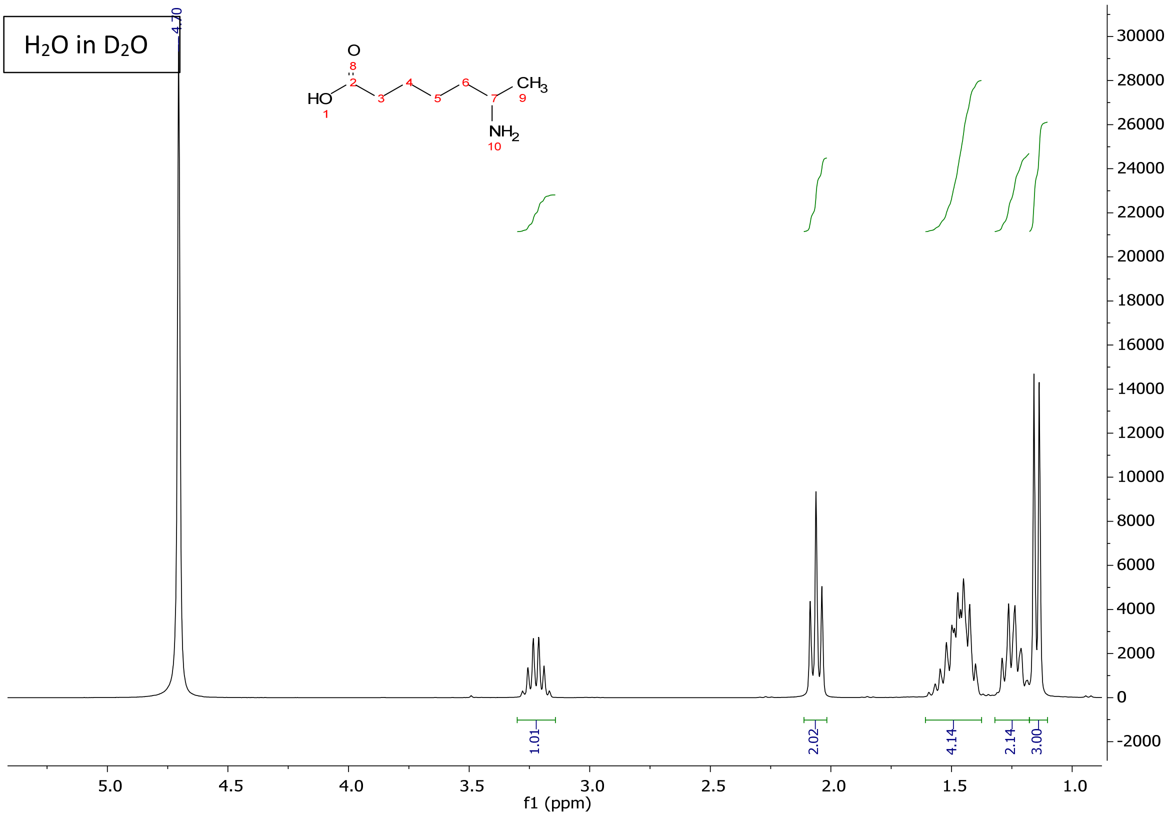

**Supplementary Figure 33**. **$^1$H-NMR spectrum of 3b**. The spectrum was recorded in $D_2O$ after purification with ion exchange chromatography on a Bruker Avance 400 MHz NMR spectrometer.

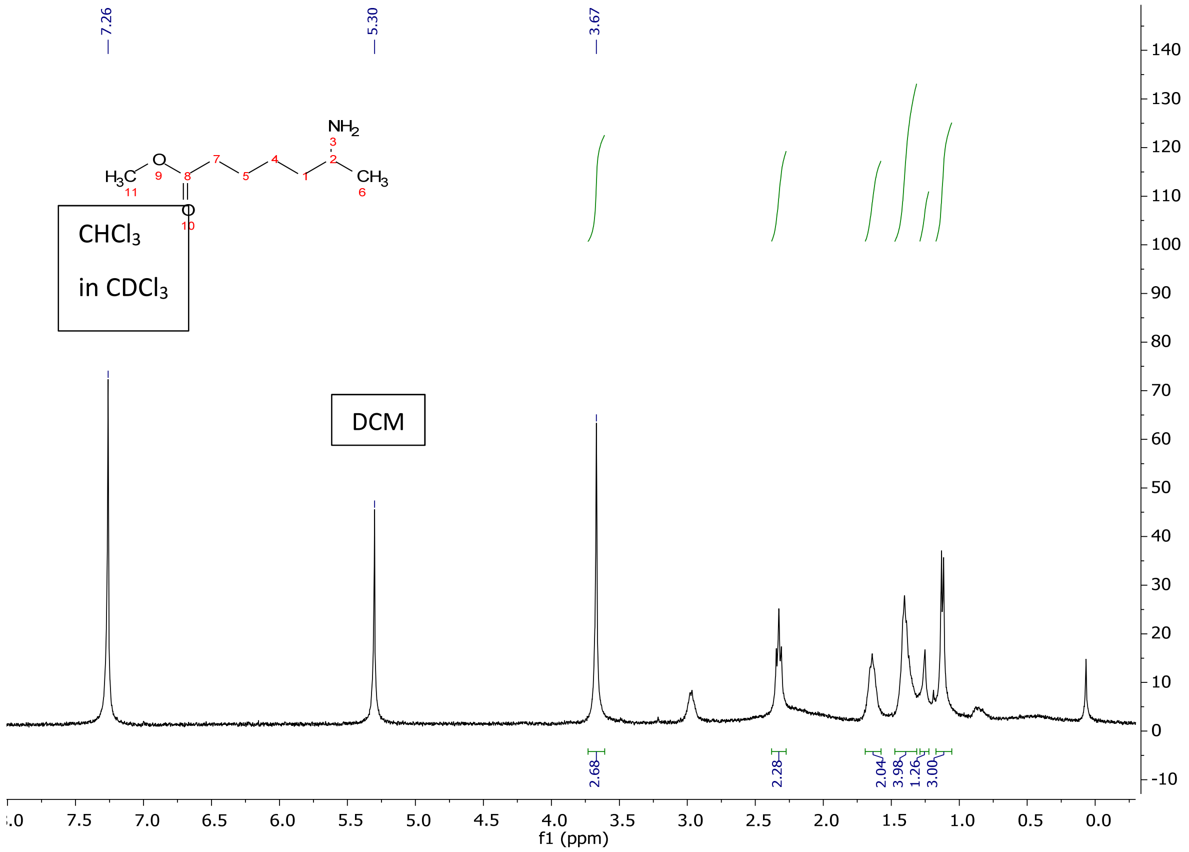


**Supplementary Figure 34**. **$^1$H-NMR spectrum of 22b.** The spectrum was recorded in $CDCl_3$, after purification of **3b** and methylation of the acid moiety using methanol and thionyl chloride as described above, on a Bruker Avance 400 MHz NMR spectrometer.

## Thermostability of LE-AmDH-v1

The thermostability (melting temperature $T_m$, defined as the temperature at which 50% of the enzyme unfolds) of LE-AmDH-v1 was determined in $HCOONH_3/NH_4$ buffer of various pH values (2 M; pH 7.5- 9.5) by differential scanning fluorometry using a Biorad-7500 QPCR machine. Each reaction mixture (20 µL) contained of SYPRO orange (Stock 20x in $qH_2O$), 2 µg of enzyme and the buffer. In selected measurements, coenzyme ($NAD^+$, 100 µM) or ligand (**8a**, 100 µM) was added. Fluorescence data were collected as a continuous standard melt curve from 20-90 °C (1% increment, hold 1 min at 20 °C and 1 min at 90 °C), using ROX for reporter and none for quencher. Also none was selected as passive reference. Three reaction replicates were prepared for every buffer tested, as well as a negative control without enzyme. Results are summarized in **Supplementary Table 17** and **Supplementary Figure 35**:

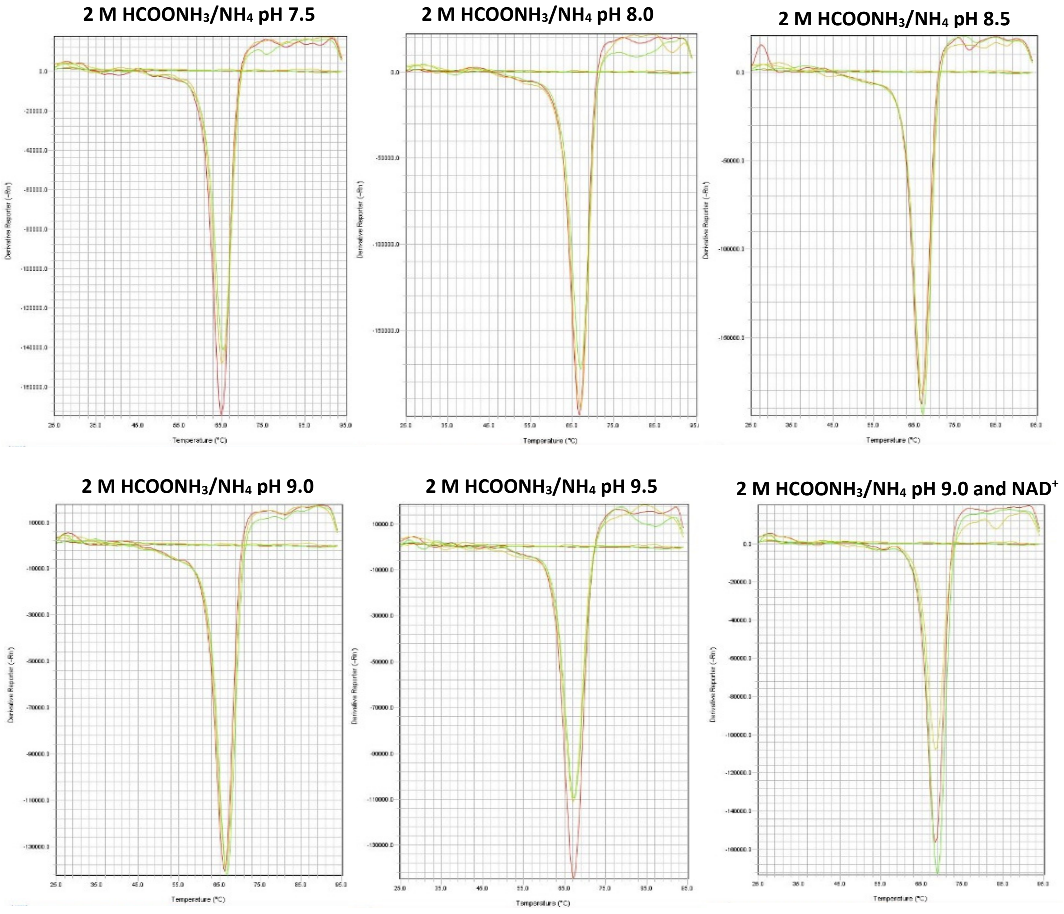


**Supplementary Figure 35**. **Thermal stability (melting temperature) of LE-AmDH-v1**. Melting curves were determined in various pH values of the ammonium formate buffer by differential scanning fluorometry.

Long-term stability studies of LE-AmDH-v1 were performed in $HCOONH_4/NH_3$ buffer (2 M, pH 9.0). In each assay, the residual enzyme activity after incubation of the enzyme at different times (up to 7 days) and temperatures (4 °C, room temp., 40 °C, 50 °C and 60 °C, respectively) was determined at 60 °C by following the oxidation of NADH (250 µM) at 360 nm (ε = 4250 $M^{-1}$ $cm^{-1}$) using a Shimadzu UV-1800 UV-vis spectrophotometer (**Supplementary Figure 36**).

A solution of **8a** (fixed final concentration 15 mM; from 500 mM main stock in DMSO, which was further diluted to 350 mM with reaction buffer) was added to preincubated reaction buffer (70 °C). The mixture was incubated further at 60 °C in the thermostatic controlled cuvette holder of the UV-vis spectrophotometer for 2 min. Then, the enzyme (4.8-5.4 µM) was added to the assay solution of **8a** and the mixture was incubated for further 30 sec at 60 °C. The reaction was started by the addition of coenzyme (final concentration 250 µM; from 50 mM main stock in 50 mM KPi buffer at pH 8.0, which was further diluted to 10 mM in reaction buffer).

The initial velocities were calculated from the linear range of the fitted trend line of the progress curve and the residual activities (100% corresponds to the enzyme after defrosting) were plotted against the incubation time. A minimum of two different purified enzyme batches were tested at every temperature.

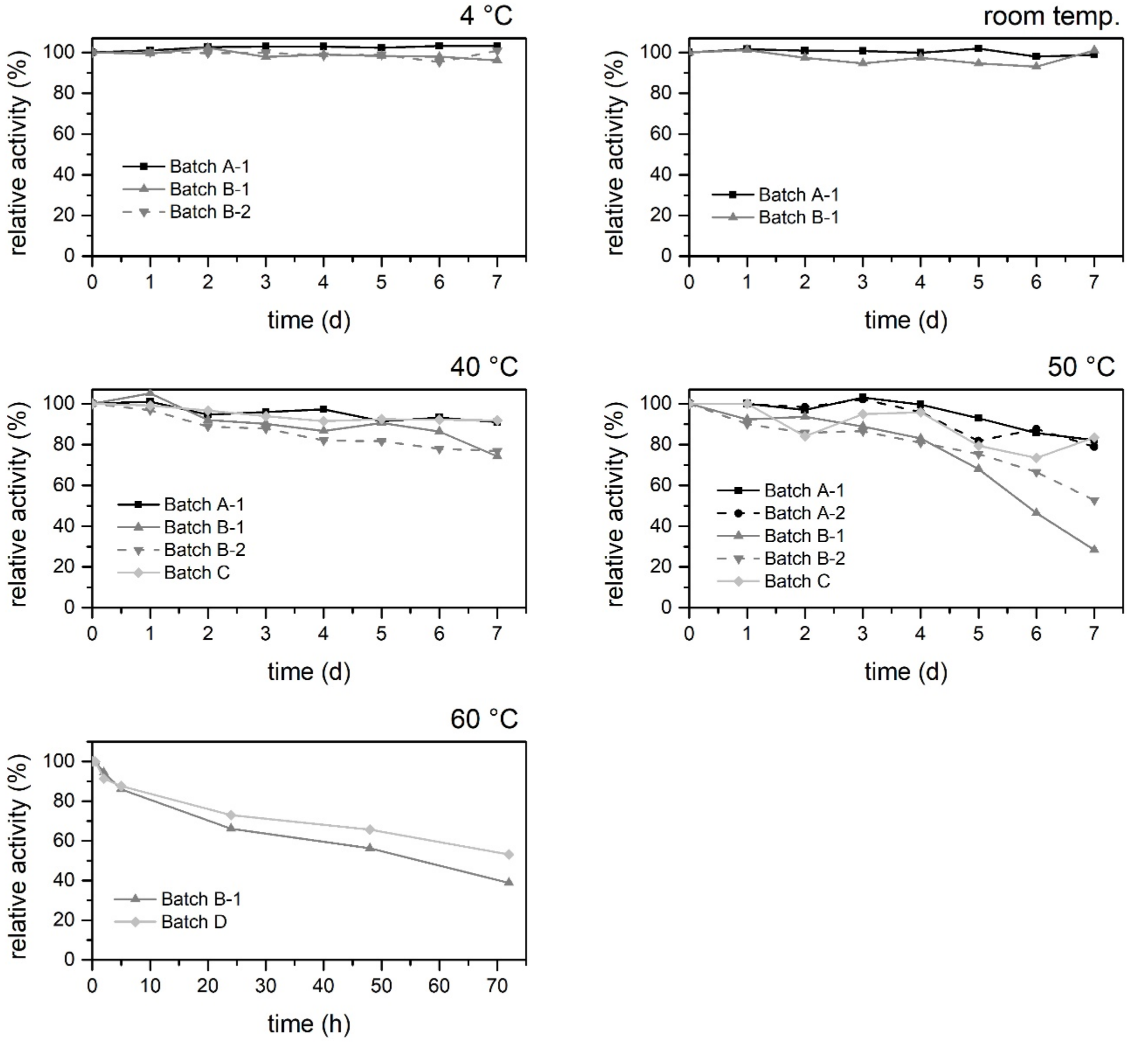


**Supplementary Figure 36**. **Long-term stability of LE-AmDH-v1.** Experiments were performed at different incubation temperatures of the enzyme, up to 7 days by following the oxidation of NADH (250 µM) at 360 nm. Source data are provided as a Source Data file.

**Steady-state kinetics and inhibition studies**

The saturation concentration of NADH for Ch1-AmDH and LE-AmDH-v1 was determined as depicted below:

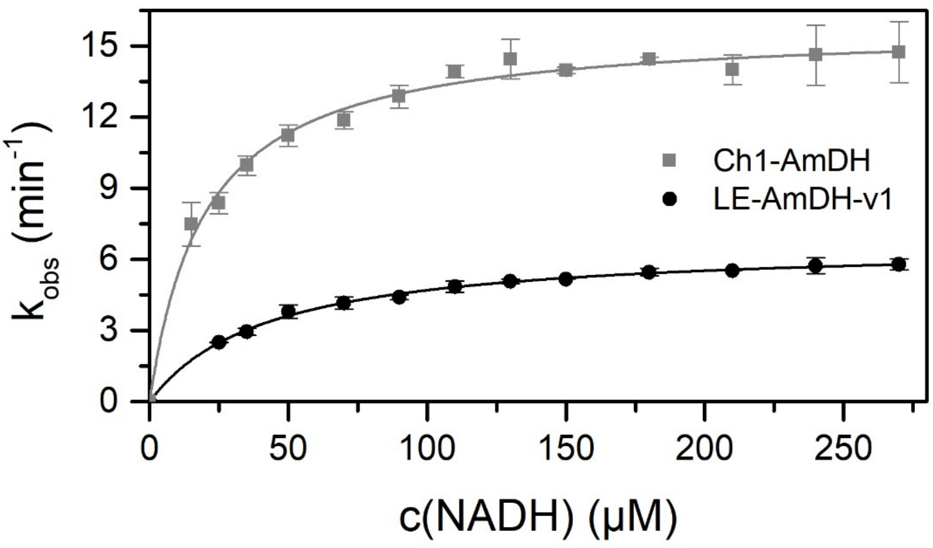


**Supplementary Figure 37**. **Michaelis-Menten plot for the determination of the saturation concentration of NADH**. The saturation concentration of NADH was determined for both LE-AmDH-v1 and Ch1-AmDH using different concentrations of NADH. Error bars represent the deviation of n=2 independent experiments using two different enzyme batches. Source data are provided as a Source Data file.

Steady-state kinetics were determined as depicted below:

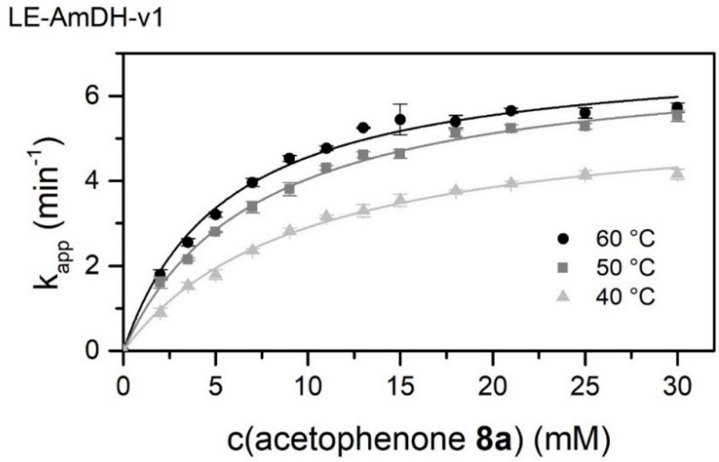


**Supplementary Figure 38. Hyperbolic fit of the reaction rates obtained for the reductive amination of 8a**. The assay was performed at different temperatures using LE-AmDH-v1, varying the concentration of **8a**. Error bars represent the deviation of n=2 independent experiments. Source data are provided as a Source Data file.

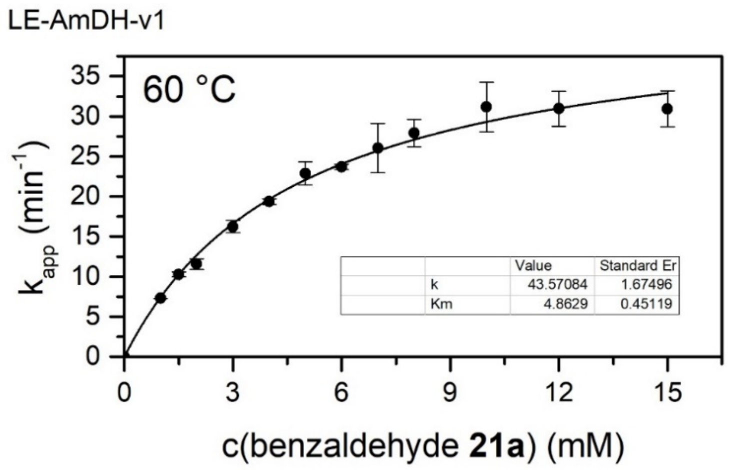


**Supplementary Figure 39. Hyperbolic fit of the reaction rates obtained for the reductive amination of 21a**. The assay was performed at 60 °C using LE-AmDH-v1, varying the concentration of **21a**. Error bars represent the deviation of n=2 independent experiments. Source data are provided as a Source Data file.

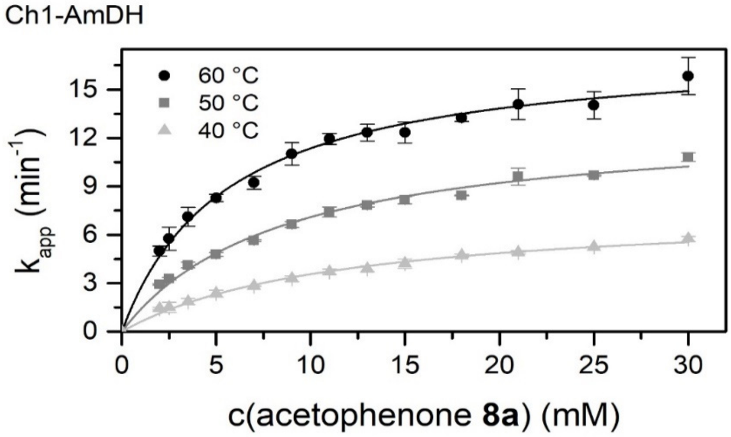


**Supplementary Figure 40. Hyperbolic fit of the reaction rates obtained for the reductive amination of 8a by Ch1-AmDH**. The assay was performed at different temperatures, varying the concentration of **8a**. Source data are provided as a Source Data file.

Inhibition studies were performed as depicted below:

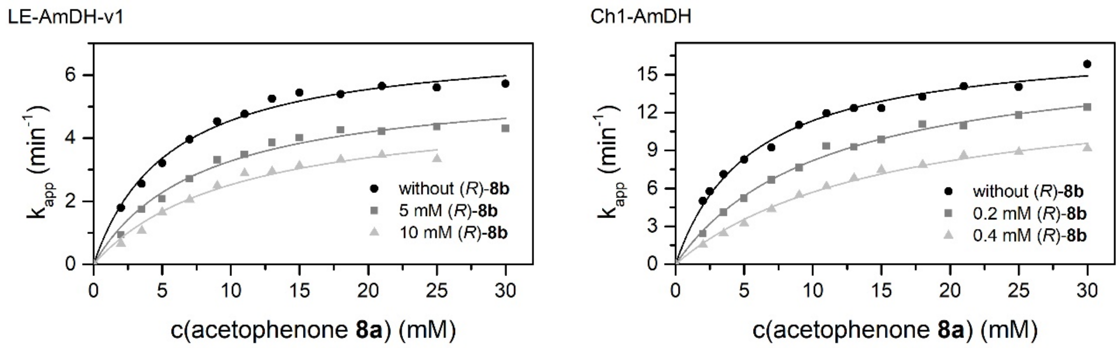


**Supplementary Figure 41. Hyperbolic fits of the reaction rates for the reductive amination of 8a in the absence and presence of (*R*)-8b.** The assay was performed for both LE-AmDH-v1 and Ch1-AmDH without and with addition (5 mM and 10 mM) of inhibitor (*R*)-**8b**. Source data are provided as a Source Data file.

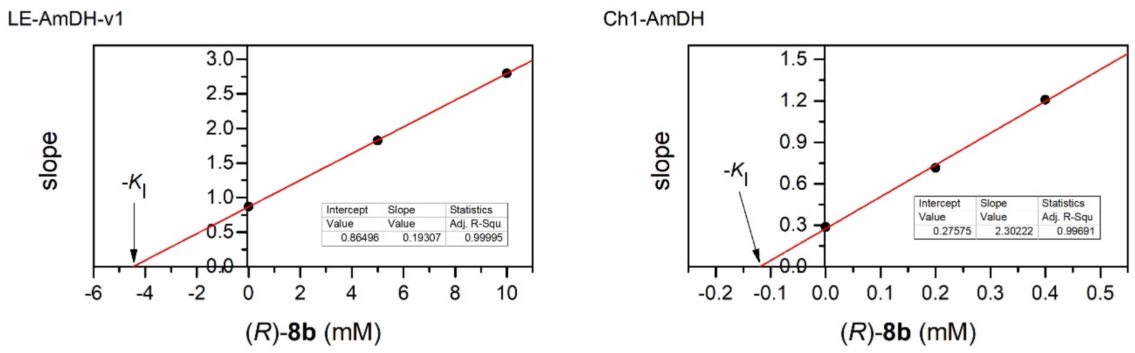


**Supplementary Figure 42. Secondary plots of the slopes obtained from the Lineweaver-Burk plots vs. concentration of inhibitor (*R*)-8b.** Ch1-AmDH shows a ~38-fold lower inhibition constant ($K_I$) than LE-AmDH-v1.

Supplementary equation 2 (reported below) was used to determine $K_M^{eff}$. $K_M^{eff}$ represents the time-averaged and effective value for the $K_M$ over the course of the reaction, thus including competitive product inhibition phenomenon. The **Supplementary equation 2** is valid if only competitive product inhibition occurs. In Equation 1, $K_M^{eff}$ is calculated as the time-averaged and effective value for the $K_M$ considering the reaction progress from the beginning until 99% conversion [30].

**Supplementary equation 2** $$K_M^{eff} = K_M \cdot 4.61 \cdot \left(1 + [S_0] \cdot \frac{0.78}{K_I}\right)$$

$K_M$ Michaelis-Menten constant, determined in steady-state kinetics in absence of inhibitor

$K_I$ Inhibitor constant, obtained from a plot of slope (double reciprocal plot) vs. concentration of inhibitor (Fig. S16)

$[S_0]$ Initial substrate concentration in biocatalytic reaction

**Analytical methods for the determination of conversions and absolute configurations**

The conversions for the reductive amination of the ketones were determined by GC using a 7890A GC system (Agilent Technologies), equipped with a FID detector using $H_2$ as carrier gas and a DB-1701 column from Agilent (30 m, 250 µm, 0.25 µm).

DB1701-30m-Method-B: constant pressure 6.9 psi, split ratio 40:1, T injector 250 °C. Temperature program: T initial 60 °C, hold 6.5 min, gradient 20 °C/min up to 100 °C; hold 1 min, gradient 20 °C/min up to 280 °C; hold 1 min.

The enantiomeric excess of the derivatised amines (as described in the main paper) was measured using a Chrompack Chiracel Dex-CB column from Agilent (25 m, 320 µm, 0.25 µm)

DEX-CB-Method-A: Constant Flow: 1.4 mL/min, split ratio 40:1, T injector 200 °C. Temperature program: T initial 100 °C, hold 2 min, gradient 1°C/min up to 130 °C; hold 5 min, gradient 10 °C/min up to 170 °C; hold 10 min, gradient 10 °C/min up to 180 °C; hold 1 min.

Amines were identified using a QP2010SE GCMS system (Shimadzu), with He as carrier gas using a DB-1701 column from Agilent (30 m, 250 µm, 0.25 µm).

DB1701-30m-Method-B: GC program parameter: linear velocity: 38.3 cm/sec, pressure 65.4 KPa, flow: 1.10 mL/min, split ratio 40:1, T injector 250 °C. Temperature program: T initial 60 °C, hold 6.5 min, gradient 20 °C/min up to 100 °C; hold 1 min, gradient 20 °C/min up to 280 °C; hold 1 min. *MS program parameter*: Ion Source Temperature: 200 °C, Detector Voltage: 0.1 Kv, Start Time: 3 min, End Time: 19.5 min, Start m/z: 43, End m/z: 600.

The RP-HPLC method for the determination of the conversion of **2a** and **3a** was carried out with a column Shim-pack GIST C18-AQ (150 mm x 4.6 mm) from Shimadzu; particle size 5 µM.

Method RP HPLC-A: constant oven temperature 40 °C; eluent composition: isocratic Milli-Q/MeOH (4%) supplemented with 0.1% trifluoroacetic acid (TFA); flow rate: 1 mL $min^{-1}$; RID-20A detector.

**Determination of ee for preparative scale reactions**

The enantiomeric excess of (*R*)-**8b** and (*R*)-**9b** (preparative scale reactions) was determined as follows. For an accurate GC-FID determination of the enantiomeric excesses of the products obtained in the preparative scale reactions catalysed by LE-AmDH-v1, 100 mM solutions of the isolated products were derivatized with DMAP in acetic anhydride.

'GC peak areas' of 16473 and 8492 were obtained for (*R*)-**8b** and (*R*)-**9b**, respectively. Commercially available *S*- and *R*- configured reference compounds were injected in the GC-FID at similar high concentrations in order to obtain 'GC peak areas' of similar intensity. It is noteworthy that — by injecting samples at such high concentration — the commercially available amines showed traces of the opposite enantiomer. Conversely, both amine samples obtained through the amination catalysed by LE-AmDH-v1 did not contain detectable traces of either (*S*)-**8b** or (*S*)-**9b** (zoom in of the overlay of the chromatograms).

Experimentally, by the use of our GC-FID system, any peak area above 2 is clearly detectable. Based on this threshold area, the *ee* values of our isolated compounds were calculated (**Supplementary Table 24**). Moreover, no trace of the ketone starting material was detected either.

## Supplementary Notes

Ketones **4a**-**12a, 14a, 17a-20a** and aldehydes **21a, 23a**, **24a** and the 2-hydroxycyclohexanone dimer used for the synthesis of **2a** were purchased from Sigma-Aldrich (Steinheim, Germany). Ketones **15a-16a** and compound **3a** were purchased from Acros Organics (Geel, Belgium) and **25a** was purchased from Fluka Chemie (Buchs, Switzerland). Finally compounds **2a** and **22a** were synthetized as described in sections 7 and 8, respectively.

Enantiomerically pure (*S*) and (*R*)-configured amines **16b, 9b-13b, 17b-20b** and **22b** were synthesized by stereoselective amination employing stereocomplementary ωTAs from HIMS-Biocat collection (University of Amsterdam) as described in section 9. Enantiomerically pure (*S*) and (*R*)-configured amines **5b** were purchased from Alfa Aesar. Racemic amine **13b** as well as enantiomerically pure compounds **8b** were purchased from TCI. Amine **21b** was purchased from Acros Organics and compound **2b** was purchased from Sigma-Aldrich.

Nicotinamide cofactor ($NAD^+$) was purchased from Melford Biolaboratories (Chelsworth, Ipswich, UK). Pyridoxal phosphate (PLP) was purchased from Alfa Aesar.Lactate dehydrogenase (LDH-101) and Glucose dehydrogenase (GDH-901) were purchased from Codexis (Redwood City, US)

## Supplementary References


1 Krieger, E., Koraimann, G. & Vriend, G. Increasing the precision of comparative models with YASARA NOVA--a self-parameterizing force field. *Proteins* **47**, 393-402, (2002).

2 Venselaar, H. et al. Homology modelling and spectroscopy, a never-ending love story. *Eur. Biophys. J.* **39**, 551-563, (2010).

3 Altschul, S. Gapped BLAST and PSI-BLAST: a new generation of protein database search programs. *Nucleic Acids Res.* **25**, 3389-3402, (1997).

4 Suzek, B. E., Huang, H., McGarvey, P., Mazumder, R. & Wu, C. H. UniRef: comprehensive and non-redundant UniProt reference clusters. *Bioinformatics* **23**, 1282-1288, (2007).

5 Jones, D. T. Protein secondary structure prediction based on position-specific scoring matrices. *J. Mol. Biol.* **292**, 195-202, (1999).

6 Laskowski, R. A., MacArthur, M. W., Moss, D. S. & Thornton, J. M. PROCHECK: a program to check the stereochemical quality of protein structures. *J. Appl. Crystallogr.* **26**, 283-291, (1993).

7 Hooft, R. W., Vriend, G., Sander, C. & Abola, E. E. Errors in protein structures. *Nature* **381**, 272, (1996).

8 Krieger, E., Darden, T., Nabuurs, S. B., Finkelstein, A. & Vriend, G. Making optimal use of empirical energy functions: force-field parameterization in crystal space. *Proteins* **57**, 678-683, (2004).

9 Yoneda, K., Fukuda, J., Sakuraba, H. & Ohshima, T. First crystal structure of L-lysine 6-dehydrogenase as an NAD-dependent amine dehydrogenase. *J. Biol. Chem.* **285**, 8444-8453, (2010).

10 Andi, B., Cook, P. F. & West, A. H. Crystal structure of the his-tagged saccharopine reductase from Saccharomyces cerevisiae at 1.7-Å resolution. *Cell Biochem. Biophys.* **46**, 17-26, (2006).

11 Johansson, E., Steffens, J. J., Lindqvist, Y. & Schneider, G. Crystal Structure of Saccharopine Reductase from Magnaporthe grisea, an Enzyme of the α-Aminoadipate Pathway of Lysine Biosynthesis. *Structure* **8**, 1037-1047, (2000).

12 Krieger, E. et al. Improving physical realism, stereochemistry, and side-chain accuracy in homology modeling: Four approaches that performed well in CASP8. *Proteins* **77**, 114-122, (2009).

13 Duan, Y. et al. A point-charge force field for molecular mechanics simulations of proteins based on condensed-phase quantum mechanical calculations. *J. Comput. Chem.* **24**, 1999-2012, (2003).

14 Berendsen, H. J. C., Postma, J. P. M., van Gunsteren, W. F. & Hermans, J. *Interaction Models for Water in Relation to Protein Hydration* in *Intermolecular Forces: Proceedings of the Fourteenth Jerusalem Symposium on Quantum Chemistry and Biochemistry Held in Jerusalem, Israel, April 13–16, 1981* (ed Bernard Pullman), 331-342 (Springer Netherlands, 1981).

15 Hooft, R. W. W., Sander, C. & Vriend, G. Positioning hydrogen atoms by optimizing hydrogen-bond networks in protein structures. *Proteins* **26**, 363-376, (1996).

16 Word, J. M., Lovell, S. C., Richardson, J. S. & Richardson, D. C. Asparagine and glutamine: using hydrogen atom contacts in the choice of side-chain amide orientation11Edited by J. Thornton. *J. Mol. Biol.* **285**, 1735-1747, (1999).

17 Canutescu, A. A., Shelenkov, A. A. & Dunbrack Jr., R. L. A graph-theory algorithm for rapid protein side-chain prediction. *Protein Sci.* **12**, 2001-2014, (2003).

18 Bussi, G., Donadio, D. & Parrinello, M. Canonical sampling through velocity rescaling. *J. Chem. Phys.* **126**, 014101, (2007).
19 Berendsen, H. J. C., Postma, J. P. M., Gunsteren, W. F. v., DiNola, A. & Haak, J. R. Molecular dynamics with coupling to an external bath. *J. Chem. Phys.* **81**, 3684-3690, (1984).
20 Hess, B., Bekker, H., Berendsen, H. J. C. & Fraaije, J. G. E. M. LINCS: A linear constraint solver for molecular simulations. *J. Comput. Chem.* **18**, 1463-1472, (1997).
21 Miyamoto, S. & Kollman, P. A. Settle: An analytical version of the SHAKE and RATTLE algorithm for rigid water models. *J. Comput. Chem.* **13**, 952-962, (1992).
22 Darden, T., York, D. & Pedersen, L. Particle mesh Ewald: An N·log(N) method for Ewald sums in large systems. *J. Chem. Phys.* **98**, 10089-10092, (1993).
23 Mutti, F. G., Knaus, T., Scrutton, N. S., Breuer, M. & Turner, N. J. Conversion of alcohols to enantiopure amines through dual-enzyme hydrogen-borrowing cascades. *Science* **349**, 1525-1529, (2015).
24 Knaus, T., Böhmer, W. & Mutti, F. G. Amine dehydrogenases: efficient biocatalysts for the reductive amination of carbonyl compounds. *Green Chem.* **19**, 453-463, (2017).
25 Heydari, M., Ohshima, T., Nunoura-Kominato, N. & Sakuraba, H. Highly Stable L-Lysine 6-Dehydrogenase from the Thermophile Geobacillus stearothermophilus Isolated from a Japanese Hot Spring: Characterization, Gene Cloning and Sequencing, and Expression. *Appl. Environ. Microbiol.* **2004**, 937-942, (2004).
26 Zhang, J., Zhang, M., Du, F.-S. & Li, Z.-C. Synthesis of Functional Polycaprolactones via Passerini Multicomponent Polymerization of 6-Oxohexanoic Acid and Isocyanides. *Macromolecules* **49**, 2592-2600, (2016).
27 Koszelewski, D., Goritzer, M., Clay, D., Seisser, B. & Kroutil, W. Synthesis of Optically Active Amines Employing Recombinant ω-Transaminases in *E.coli* Cells. *ChemCatChem* **2**, 73-77, (2010).
28 Mutti, F. G., Fuchs, C. S., Pressnitz, D., Sattler, J. H. & Kroutil, W. Stereoselectivity of Four (R)-Selective Transaminases for the Asymmetric Amination of Ketones. *Adv. Synth. Catal.* **353**, 3227-3233, (2011).
29 Mutti, F. G., Fuchs, C. S., Pressnitz, D., Turrini, N. G., Sattler, J. H., Lerchner, A., Skerra, A. & Kroutil, W. Amination of Ketones by Employing Two New (S)-Selective ω-Transaminases and the His-Tagged ω-TA from Vibrio fluvialis. *Eur. J. Org. Chem.* **2012**, 1003-1007, (2012).
30 Fox, R. J. & Clay, M. D. Catalytic effectiveness, a measure of enzyme proficiency for industrial applications. *Trends Biotechnol.* **27**, 137-140, (2009).
